# Identification of biomarkers for the diagnosis of type 2 diabetes mellitus with metabolic associated fatty liver disease by bioinformatics analysis and experimental validation

**DOI:** 10.3389/fendo.2025.1512503

**Published:** 2025-01-28

**Authors:** Guiling Wu, Sihui Wu, Tian Xiong, You Yao, Yu Qiu, Liheng Meng, Cuihong Chen, Xi Yang, Xinghuan Liang, Yingfen Qin

**Affiliations:** ^1^ Department of Endocrinology, The First Affiliated Hospital of Guangxi Medical University, Nanning, Guangxi, China; ^2^ Guangxi Key Laboratory of Precision Medicine in Cardio-Cerebrovascular Diseases Control and Prevention, First Affiliated Hospital, Guangxi Medical University, Nanning, Guangxi, China; ^3^ Department of Geriatric Endocrinology and Metabolism, First Affiliated Hospital, Guangxi Medical University, Nanning, Guangxi, China; ^4^ Guangxi Clinical Research Center for Cardio-Cerebrovascular Diseases, Nanning, Guangxi, China

**Keywords:** secreted protein, metabolic associated fatty liver disease, type 2 diabetes mellitus, TNFRSF1A, SERPINB2

## Abstract

**Background:**

Type 2 diabetes (T2DM) combined with fatty liver is a subtype of metabolic fatty liver disease (MAFLD), and the relationship between T2DM and MAFLD is close and mutually influential. However, the connection and mechanisms between the two are still unclear. Therefore, we aimed to identify potential biomarkers for diagnosing both conditions.

**Methods:**

We performed differential expression analysis and weighted gene correlation network analysis (WGCNA) on publicly available data on the two diseases in the Gene Expression Omnibus database to find genes related to both conditions. We utilised protein–protein interactions (PPIs), Gene Ontology, and the Kyoto Encyclopedia of Genes and Genomes to identify T2DM-associated MAFLD genes and potential mechanisms. Candidate biomarkers were screened using machine learning algorithms combined with 12 cytoHubba algorithms, and a diagnostic model for T2DM-related MAFLD was constructed and evaluated.The CIBERSORT method was used to investigate immune cell infiltration in MAFLD and the immunological significance of central genes. Finally, we collected whole blood from patients with T2DM-related MAFLD, MAFLD patients and healthy individuals, and used high-fat, high-glucose combined with high-fat cell models to verify the expression of hub genes.

**Results:**

Differential expression analysis and WGCNA identified 354 genes in the MAFLD dataset. The differential expression analysis of the T2DM-peripheral blood mononuclear cells/liver dataset screened 91 T2DM-associated secreted proteins. PPI analysis revealed two important modules of T2DM-related pathogenic genes in MAFLD, which contained 49 nodes, suggesting their involvement in cell interaction, inflammation, and other processes. TNFSF10, SERPINB2, and TNFRSF1A were the only coexisting genes shared between MAFLD key genes and T2DM-related secreted proteins, enabling the construction of highly accurate diagnostic models for both disorders. Additionally, high-fat, high-glucose combined with high-fat cell models were successfully produced. The expression patterns of TNFRSF1A and SERPINB2 were verified in patient blood and our cellular model. Immune dysregulation was observed in MAFLD, with TNFRSF1A and SERPINB2 strongly linked to immune regulation.

**Conclusion:**

The sensitivity and accuracy in diagnosing and predicting T2DM-associated MAFLD can be greatly improved using SERPINB2 and TNFRSF1A. These genes may significantly influence the development of T2DM-associated MAFLD, offering new diagnostic options for patients with T2DM combined with MAFLD.

## 1 Introduction

Metabolic dysfunction associated fatty liver disease (MAFLD) is a chronic condition characterized by hepatic steatosis, along with at least one of the following: overweight/obesity, type 2 diabetes (T2DM), or metabolic dysfunction. It was proposed in 2020 as an alternative to the traditional term non-alcoholic fatty liver disease (NAFLD) (1). Of all adult diseases, metabolic associated fatty liver disease (MAFLD) affects around one-third of individuals globally (2). In its more advanced phases, MAFLD can lead to hepatic fibrosis and hepatocellular cancer, in addition to steatohepatitis (3–5). As one of the fastest liver-related disorders in incidence and mortality (6), effectively managing MAFLD is a major global public health concern (7).

There is a close relationship between MAFLD and T2DM, with both being mutually causal and forming a vicious cycle. According to epidemiological studies, patients with MAFLD are nearly twice as likely to acquire T2DM as the general population, regardless of obesity or other prevalent metabolic risk factors (8). AND the probability of MAFLD in T2DM patients is 50%~75% higher than that of the normal population (9).As a metabolic syndrome characterised by hyperglycaemia, insulin resistance, and impaired insulin secretion, T2DM induces an increase in lipolysis, which in turn causes the liver to absorb excessive amounts of free fatty acids (10). Simultaneously, excessive intrahepatic lipid deposition inhibits intrahepatic insulin signalling, resulting in a decrease in glycogen synthesis and an increase in gluconeogenesis, causing fluctuations in the body’s insulin and glucose levels (11). Ultimately, this creates a vicious cycle. Once fatty liver appears in T2DM patients, it can be diagnosed as MAFLD. The combination of MAFLD and T2DM not only increases the cardiovascular complications risk for type 2 diabetes patients, but may also lead to severe liver disease (12). Simultaneously, when the blood glucose level of MAFLD patients progresses to diabetes, the worsening glycaemic condition accelerates the progression of MAFLD, not only in terms of increased fatty infiltration but also in raising the risk of cardiovascular disease, the rate at which liver-related disorders result in death, and the rate at which hepatic fibrosis progresses—all of which impact patients’ quality of life (13, 14). Consequently, it is important to reveal the comorbid genes and mechanisms of MAFLD and T2DM.

Despite increasing evidence of their relationship, the primary molecules and underlying mechanisms of the substantial correlation between MAFLD and T2DM remain unknown. Therefore, this study utilised bioinformatics and machine learning techniques to explore the pathogenic genes and diagnostic biomarkers of T2DM-associated MAFLD, intending to offer a foundation for the clinical diagnosis of patients with T2DM and MAFLD, as well as research into the pathogenic mechanisms.

## 2 Materials and methods

### 2.1 Data collection and preliminary processing

The Gene Expression Omnibus (GEO) database is a publicly available functional genomics resource that provides valuable high-throughput microarray and next-generation sequencing data on functional genomes (15) Using the GEOquery package, we downloaded two MAFLD datasets (GSE89632 and GSE66676) and two type 2 diabetes datasets (GSE95849 and GSE23343) (16), which are displayed in **Table 1**. The limma software was utilised to normalise and standardise the data, with the exception of the GSE66676 dataset, which served as the test set in this investigation (17).

**Table 1:** Descriptive statistics of the GEO datasets.

GEO accession	Platform	Origin	Sample		Species
			Control	MAFLD	
GSE89632	GPL14951	Liver	24	39	Homo sapiens
GSE66676	GPL6244	Liver	24	20	Homo sapiens
GEO accession	Platform	Origin	Sample		Species
			Control	T2DM	
GSE95849	GPL22448	PBMC	6	12	Homo sapiens
GSE23343	GPL570	Liver	7	10	Homo sapiens

### 2.2 Weight gene correlation network analysis to construct gene co-expression networks and identify functional modules

WGCNA was performed using the GSE89632 dataset to find coexisting gene modules, investigate the connections between gene networks and phenotypes, and examine the network’s core genes (18). After screening the soft threshold power levels, a value of 4 was determined to be the ideal threshold. Subsequently, scale-free networks and topology matrices were built using the soft thresholds, and hierarchical clustering was conducted. A minimum of fifty genes was required for a module to be considered, and each module was assigned a distinct colour label. As a result, 24 modules were obtained using Pearson’s correlation analysis to determine the relationships between the modules and clinical features. Lastly, blue and brown modules—the ones with the strongest positive and negative module-trait relationships—were filtered.

### 2.3 Screening for differential genes between disease and health groups

Background correction, normalization, and gene symbol conversion were applied to the MAFLD dataset and the T2DM dataset (GSE95849 and GSE23343). The R package DESeq2 and limma was employed for differential gene analysis of GEO microarray data in the normal and disease groups (19). The thresholds for identifying differential genes were |logFC| >1 and P < 0.05 for the control versus MAFLD disease dataset, and |logFC| >0.5 and P < 0.05 for the normal versus diabetes disease groups. Subsequently, the ‘ggplot2’ and ‘pheatmap’ tools in R software were used to visualize the expression patterns of differentially expressed genes (DEGs) as volcano plots and heat maps, respectively.

### 2.4 Secreted protein acquisition

The Human Protein Atlas database (https://www.proteinatlas.org/) provided the secreted protein data (20). SPOCTOPUS predicted a total of 3947 genes encoding secreted proteins.

### 2.5 Acquisition of shared genes

The overlapping genes from the DEGs and WGCNA modules in GSE89632 were defined as MAFLD-associated shared genes, while the intersections of DEGs with secreted protein genes within the GSE95849 and GSE23343 datasets, respectively, were identified as T2DM-associated secreted proteins that are shared with DEGs, both visualized through Venn diagrams. To explore the interactions between T2DM-associated secretory proteins and key MAFLD genes, a protein–protein interaction (PPI) network connecting T2DM and MAFLD was established based on the STRING database (https://www.stringdb.org), with a median confidence score of > 0.4 (21). Subsequently, the PPI network was visualised using Cytoscape software (version 3.9.1) (22). To identify important modules, we also conducted molecular complex detection (MCODE) using Cytoscape. The top two scoring modules associated with T2DM and MAFLD genes were chosen for enrichment analysis.

### 2.6 Functional enrichment analysis

We performed Kyoto Encyclopedia of Genes and Genomes (KEGG) annotation and Gene Ontology (GO) annotation on the genes utilising the R package ‘clusterProfiler’ to investigate the distinct processes and biological functions of T2DM-associated MAFLD-causing genes. Pathway enrichment analyses for Hs.eg.db with adj.p < 0.05 were deemed significantly enriched (23). Ring and bubble plots were also used to display the functional enrichment analysis results.

### 2.7 Screening pivotal genes according to machine learning and PPI networks

We used the overlapping genes between secreted proteins and DEGs from the GSE95849 and GSE23343 datasets as the T2DM-associated MAFLD shared genes in order to identify candidate biomarkers and construct a diagnostic model for T2DM-associated MAFLD. Initially, we employed the least absolute shrinkage and selection operator (LASSO) algorithm, a data-mining technique that applies an L1 penalty (lambda) to set the coefficients of less important variables to zero, filtering out important variables to construct the best classification model (24). Thereafter, we used support vector machine recursive feature elimination (SVM-RFE) analysis to identify the optimal core genes by discarding the feature vectors generated by the SVM (25) and applied the Random Forest (RF) algorithm, which integrates multiple trees through integrative learning for improved accuracy and employs the ‘Random Forest’ software package to narrow down candidate biomarkers (26). This approach helps establish the intersection of the three machine learning algorithms. Additionally, the cytoHubba plug-in is frequently used, much like machine learning algorithms, for the identification of key genes (27). For this reason, in this study, a PPI network was constructed once more from the STRING database and visualised using Cytoscape. Next, 12 cytoHubba plug-in algorithms were used to assess differential genes, and lastly, the top 10 genes from each algorithm as intersections were visualised using the ImageGP platform (28). Ultimately, all of the genes that were discovered using both techniques were recognised as hub genes.

### 2.8 Expression of T2DM-related MAFLD hub genes in the MAFLD dataset

The expression levels of core genes were determined using two datasets: the validation dataset GSE66676 and the training set GSE89632.

### 2.9 Construction of nomograms and evaluation of predictive models for diagnostic markers

Using the ‘rms’ software program, alignment diagrams based on the three pivotal genes were created (29). The area under the subject’s receiver operating characteristic (ROC) curve was plotted to evaluate the effectiveness of each pivotal gene and alignment diagram in the diagnosis of MAFLD. Furthermore, ROC curves were generated to ascertain whether the diagnosis of MAFLD was aided by decision-making based on the nomograms. To evaluate the predictive effectiveness of the alignment diagram for T2DM-related MAFLD, calibration curves and decision curve analysis (DCA) were conducted sequentially, with the aforementioned validation performed in the validation set.

### 2.10 Analysis of immune infiltration

Using the ‘CIBERSORT’ software tool, the degree of immune cell infiltration was evaluated in relation to MAFLD gene expression profiles (30). The ‘ggplot2’ software tool was utilized to generate a bar graph representing the percentage and quantity of immune infiltration for each sample. The proportions of 22 immune cells in MAFLD samples and control liver tissue samples were compared using the Wilcoxon test. A statistically significant difference was defined as P < 0.05, and the results were displayed using stacked histograms from the ‘ggplot2’ package. Subsequently, the ‘corrplot’ software was used to investigate the associations among the 22 infiltrating immune cells (31). Lastly, Spearman’s rank correlation coefficient was employed to assess the relationship between the expression of diagnostic biomarkers and the amount of infiltrating immune cells. The results showed that the association was statistically significant at P < 0.05.

### 2.11 Specimen collection

The study was approved by the Ethics Committee of the First Affiliated Hospital of Guangxi Medical University. All blood samples were collected from the Department of Endocrinology. Furthermore, the inclusion criteria for non-T2DM-related MAFLD group (hereinafter referred to as the MAFLD group) in this study were those diagnosed with hepatic steatosis by ultrasound examination, while also meeting the criteria of being overweight or obese or having metabolic abnormalities (1), excluding patients with T2DM. Conversely, patients diagnosed with hepatic steatosis through ultrasound examination and meeting T2DM criteria are classified as the T2DM-related MAFLD group. The diagnosis of T2DM is based on the diabetes diagnostic criteria recommended by the World Health Organization (WHO) in 1999.

### 2.12 Experimental materials

DMEM medium was purchased from WISENT (Canada), and fetal bovine serum was procured from ViaCell (Shanghai, China). Penicillin-streptomycin-amphotericin B antibodies were obtained from Solepol (Beijing, China), while sodium palmitate was sourced from Xi’an Kunchuang Science and Technology Development Co Ltd (Xi’an, China). The CCK-8 reagent was acquired from Xin Saimai (Suzhou, China), and SERPINB2, TNFSF10, and TNFRSF1A antibodies were obtained from Proteintech (Wuhan, China). The goat anti-rabbit antibody from SAB Signalway Antibody (China) was used for Western blotting. The Western Lightning™ Plus-ECL Enhanced Chemiluminescent Substrate Detection Kit was purchased from HYCEZMBIO (Wuhan, China), and the goat anti-rabbit secondary antibody for immunofluorescence was obtained from Servicebio (Wuhan, China). The modified Oil Red O staining kit was acquired from Biyuntian (China), and the triglyceride (TG) assay kit was sourced from Nanjing Jianjian Bioengineering Institute (Nanjing, China). TRIzol (Takara, Tokyo, Japan), RT SuperMix for qPCR, and SYBR qPCR Master Mix (Ruizhen Bio, Guangzhou, China) experimental reagents were utilised, while the QuantStudio 3/5 Real-Time PCR Software System (Thermo Fisher Scientific, USA) and eBlot^®^ Electronic Compression Imager (Shanghai, China) were employed for RT-PCR and Western blotting.

### 2.13 Cell model construction and cell culture

The HepG2 cells were obtained from the cell bank of the Chinese Academy of Sciences’ Typical Culture Preservation Committee. The cells were cultivated in a cell culture incubator at 37°C with 5% CO2 using a complete medium (DMEM containing 1% penicillin-streptomycin-amphotericin B triple antibody, 10% fetal bovine serum, and 5.5 mmol/L glucose). In this experiment, 5.5 mmol/L of glucose was designated as the low glucose concentration, while 25 mmol/L was considered the high glucose concentration (32). HepG2 cells were injected into 96-well plates at a density of 1.5 × 10^4^ cells/well once their growth density had reached 70–80%. They were then incubated until the cells reached 50% confluence, at which point they were treated with different concentrations of sodium palmitate (PA) medium, with a glucose concentration of 25 mmol/L, for 48 hours. Following this, the CCK8 assay was conducted to determine appropriate sodium palmitate concentrations. In a 6-well plate, approximately 1 × 105 cells were cultured for a full day after infection. To treat the cells for 48 hours, the appropriate concentration of sodium palmitate was added to the complete medium containing either 5 mmol/L or 25 mmol/L glucose. Both the T2DM combined MAFLD model (HGHF group) and the MAFLD model (LGHF group) were established. Fat accumulation levels were assessed using a triglyceride kit and Oil Red O staining, with a control group (LGLF group) consisting of the complete culture medium mixed with an equal volume of control solvent.The GPO-PAP method was employed to determine the TG content using a TG assay kit. The triglyceride test reagents and cell homogenate were added to a 96-well plate according to the instructions, mixed, and incubated at 37°C for 10 minutes. Subsequently, the optical density (OD) of each well was measured using a microplate reader (546 nm). The following formula was used to calculate the TG content: TG content = (sample OD value - blank OD value)/(calibration sample OD value - blank OD value) * (calibration sample concentration/sample protein concentration).

### 2.14 Validation of hub gene expression between the control, MAFLD, and T2DM-associated MAFLD groups

Using TRIzol reagent, total RNA was isolated from cultivated cells, and RT SuperMix was used to reverse transcribe the resulting cDNA for qPCR. cDNA that had been separated was kept at -80°C. Using the QuantStudio 3/5 Real-Time PCR Software System and SYBR qPCR Master Mix, the target genes were amplified and identified. The 2-ΔΔCt technique was used to repeat each experiment and calculate the relative mRNA expression. Primers as follows: TNFRSF1A: Forward: 5’-GTA TCG CTA CCA ACG GTG GAA GTC-3 and Reverse: 5’-TGA AGC CTG GAG TGG GGA CTG AAG-3’; TNFSF10: Forward: 5’-TTA CCA ACG AGC TGA AGC AGA TGC-3’ and Reverse: 5’-GCT GAC GGG AGT TGC CAC TTG AC-3’; SERPINB2: Forward: 5’-GCT TCC AGA TGA AAT TGC CGA TGT G-3’ and Reverse: 5’-TGT CTT TGC TGG TCC ACT TGT TGA G-3’;β-ACTIN: Forward: 5’-GGC CAA CCG CGA GAA GAT GAC-3’ and Reverse: 5’-GGA TAG CAC AGC CTG GAT AGC AAC-3’. Using conventional techniques, total proteins from HepG2 cells were extracted, and a BCA protein assay kit was utilised to measure protein concentrations. An SDS-PAGE gel containing 40 μg of protein was prepared and electrophoresed for 1.5 hours at 120 V. After separation, the proteins were transferred onto a PVDF membrane and quickly rotated for 45 minutes at 350 mA. The membrane was then blocked for one hour at room temperature in a buffer containing five percent skimmed dry milk. Next, it was incubated for one hour at room temperature with a primary antibody (1:1000), washed with Tris Buffered Saline with Tween-20, and left overnight at 4°C in a corresponding primary antibody solution. The membranes were treated with a secondary antibody (1:15,000) for one hour after being cleaned with TBST the following day. Tubulin served as a control for supersampling. An eBlot^®^ Electronic Compression Imager (Shanghai, China) was used for signal identification. The software ImageJ 1.41 was utilised to compute optical density.

### 2.15 Statistical analysis

The R programming language, version 4.0.2, was used for all statistical analyses and data computations. Statistical analysis was performed using GraphPad Prism 9.5.0. The Mann–Whitney U-test, also known as the Wilcoxon rank sum test, was employed to examine differences between non-normally distributed variables, while the independent Student t-test was used to determine the statistical significance of normally distributed variables. In the HepG2 cell model, multiple comparisons were performed using one-way ANOVA followed by Tukey’s multiple comparison test. In human blood, multiple comparisons were performed using the Kruskal-Wallis test followed by Dunn’s multiple comparisons test. All statistical P-values were two-sided, with a P-value of less than 0.05 regarded as statistically significant.

## 3 Result

### 3.1 Identification of MAFLD-related genes

**Figure 1:**
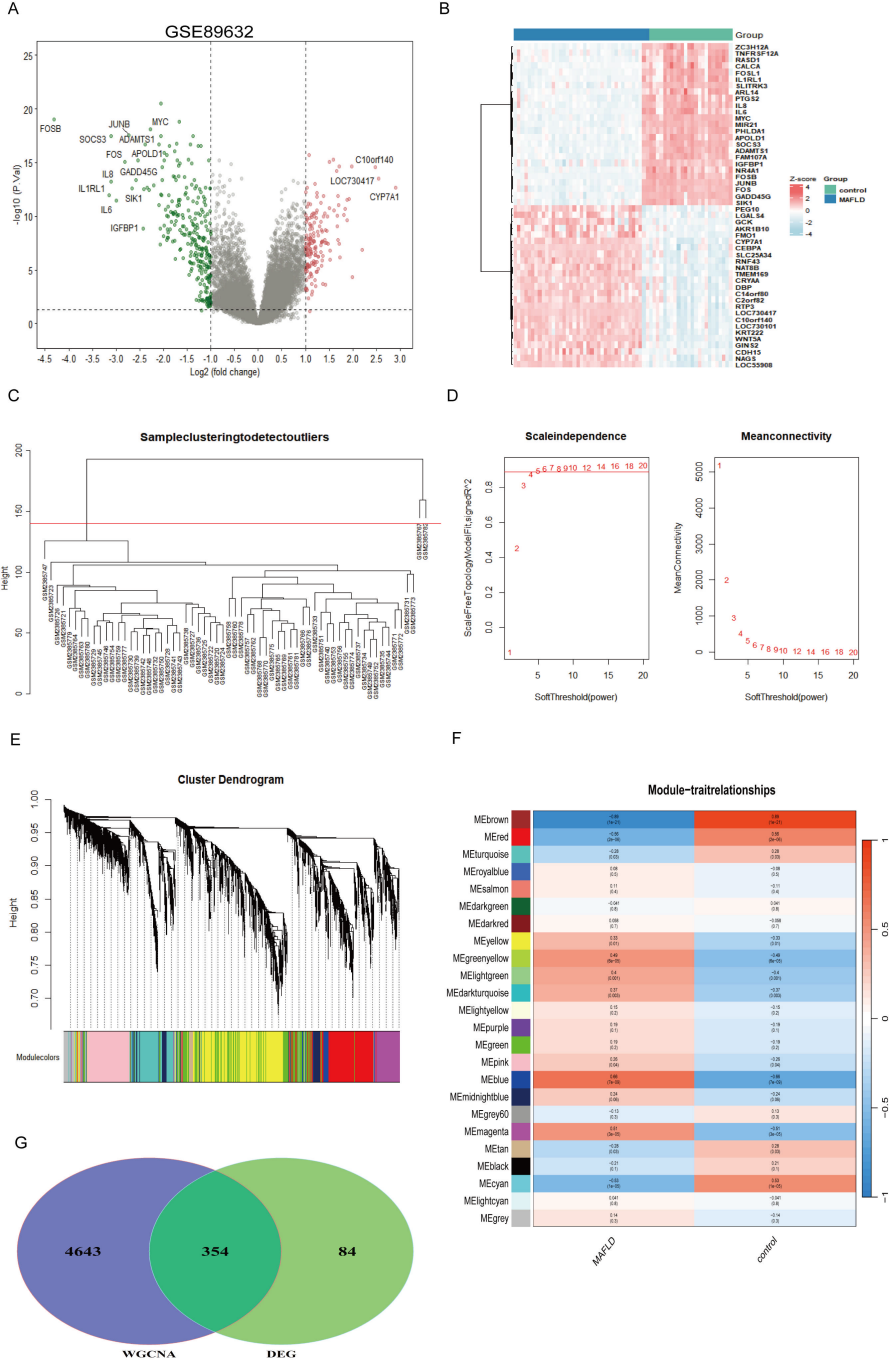
Analysis of differentially expressed genes in the MAFLD dataset.**(A)** Volcano plot of differentially expressed genes. The red node indicates up-regulated genes with a P-value < 0.05 and logFC > 1.0; the green node indicates down-regulated genes with the P-value < 0.05 and logFC < -1.0. **(B)** Heatmap of 25 up-regulated genes and 25 down-regulated genes. **(C)** Euclidean distance clustering tree of samples. The red line of the dataset of MAFLD was the tangent line of outlier detection. Samples cut by this line are considered outliers. **(D)** The scale-free fitting index (β) of each soft threshold power and the average connectivity of each soft threshold power were analysed, with a power value of 4 identified as the most suitable. **(E)** Cluster dendrogram of module characteristic genes: each colour represents a module, while grey represents genes not included in any of the modules. **(F)** Heatmap illustrating the correlation between module characteristic genes and MAFLD disease states. The blue module displayed the largest positive correlation with MAFLD, while the brown module showed the largest negative correlation. **(G)** WGCNA was integrated with DEG analysis to obtain MAFLD-related shared genes.

When |logFC| > 1 and P < 0.05 was used as the threshold, analysis of the differences between MAFLD and control liver tissue samples revealed a total of 438 DEGs, comprising 168 up-regulated genes and 270 down-regulated genes (**Figures 1A, B**). Based on average connectivity and scale independence, WGCNA analysis was utilized to determine which gene modules in MAFLD samples were the most relevant. A soft threshold power was then chosen, allowing the construction of scale-free networks and topological matrices (**Figure 1C**). A total of twenty-four modules were produced. **Figure 1D** displays the modules’ clustering dendrogram, while **Figure 1E** illustrates the association between GCMs and MAFLD samples. According to the findings, the blue and brown modules were selected as key modules for further analysis, as the results indicated that the blue module had the strongest positive association (r = 0.66, P = 7e-09) and the largest negative correlation (r = 0.89, P = 1e-21) with MAFLD (**Figure 1F**). Following the identification of 4997 key genes significantly associated with MAFLD in the blue and brown modules, the DEGs in MAFLD patient specimens were intersected with the key genes from the WGCNA to identify the key genes in MAFLD. This resulted in the identification of 354 causative genes (**Figure 1G**), which will be the focus of our upcoming analyses.

### 3.2 Identification of differentially expressed secreted proteins in type 2 diabetes mellitus

**Figure 2:**
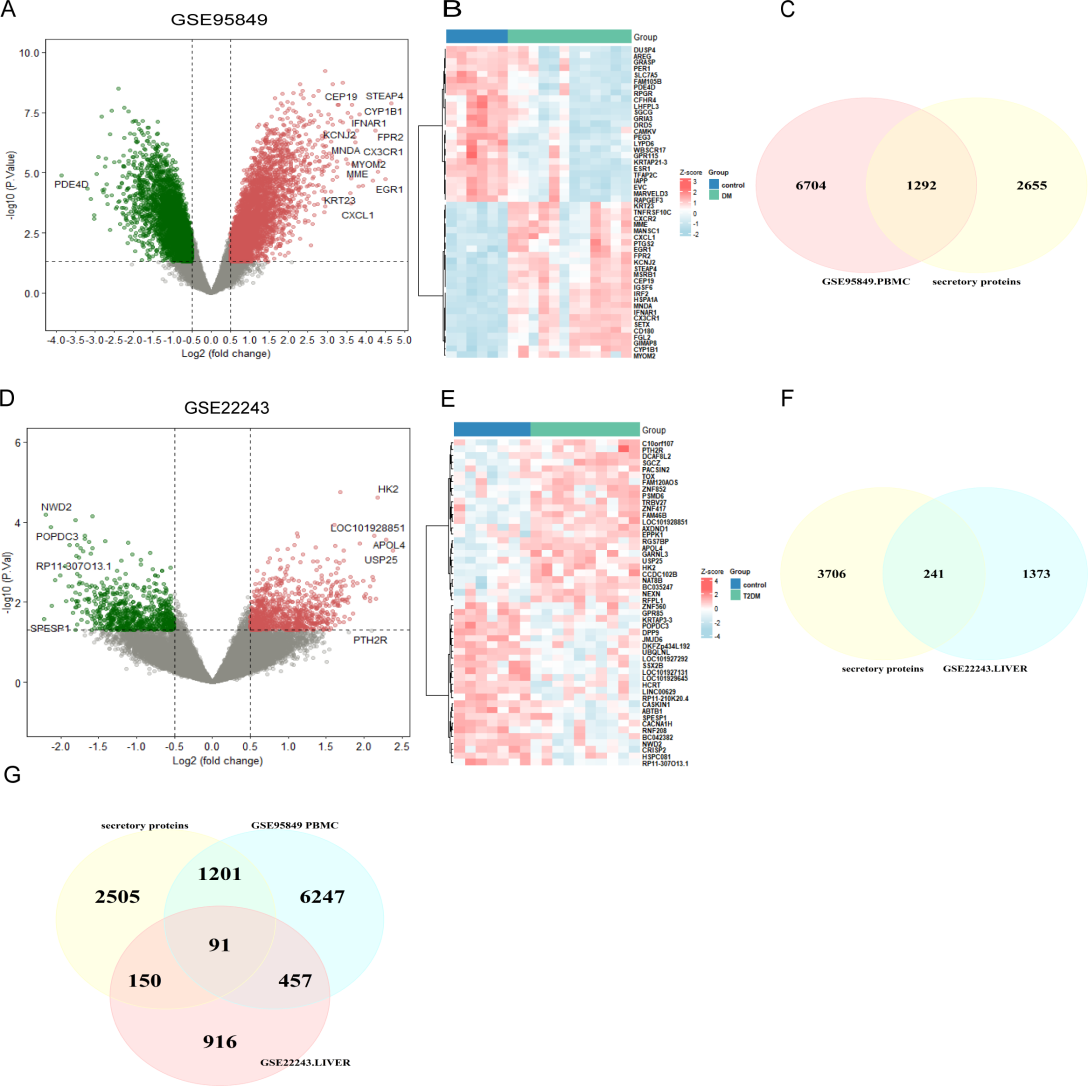
Screening of T2DM-related secreted protein genes. **(A)** Volcano plot of DEGs in GSE95849. **(B)** Heatmap of the 50 most significantly differentially expressed genes in GSE95849. **(C)** DEGs identified in GSE95849 were compared with secreted protein genes from the Human Protein Atlas to identify 1,292 T2DM-PMBC-secretory proteins. **(D)** Volcano plot of DEGs in GSE22243. **(E)** Heatmap of the 50 most significantly differentially expressed genes in GSE22243. **(F)** DEGs identified in GSE22243 were compared with secreted protein genes from the Human Protein Atlas to identify 241 T2DM-associated liver secretory proteins. **(G)** Venn diagram showing the co-secreted protein genes in both PBMC and liver datasets from T2DM patients. DEGs, differentially expressed genes; PBMCS, peripheral blood mononuclear cells (PBMCs).

It is well established that there is a causal relationship between T2DM and MAFLD, with MAFLD potentially accelerating the onset and progression of T2DM (13, 14). We first examined the expression profiles of T2DM-associated peripheral blood mononuclear cells (PBMCs) and T2DM liver tissues using the GEO database to identify the genes that contribute to T2DM. Using the thresholds of |logFC| > 0.5 and P < 0.05, we screened 1,614 causative genes from T2DM liver tissues and 7,996 causative genes from T2DM-PBMCs. Given the potential significance of secreted proteins in the pathogenesis of T2DM and MAFLD (33, 34), T2DM liver DEGs and T2DM-PBMC DEGs were intersected with the Human Protein Atlas to identify 241 and 1,292 secretory proteins, respectively (**Figures 2A–F**). Among the secreted T2DM-associated proteins, 91 were shared by the two sets of proteins (**Figure 2G**). The remaining 1,442 genes were identified as T2DM-related secreted proteins for further analysis.

### 3.3 PPI and functional enrichment analysis of T2DM-related MAFLD causative genes

**Figure 3:**
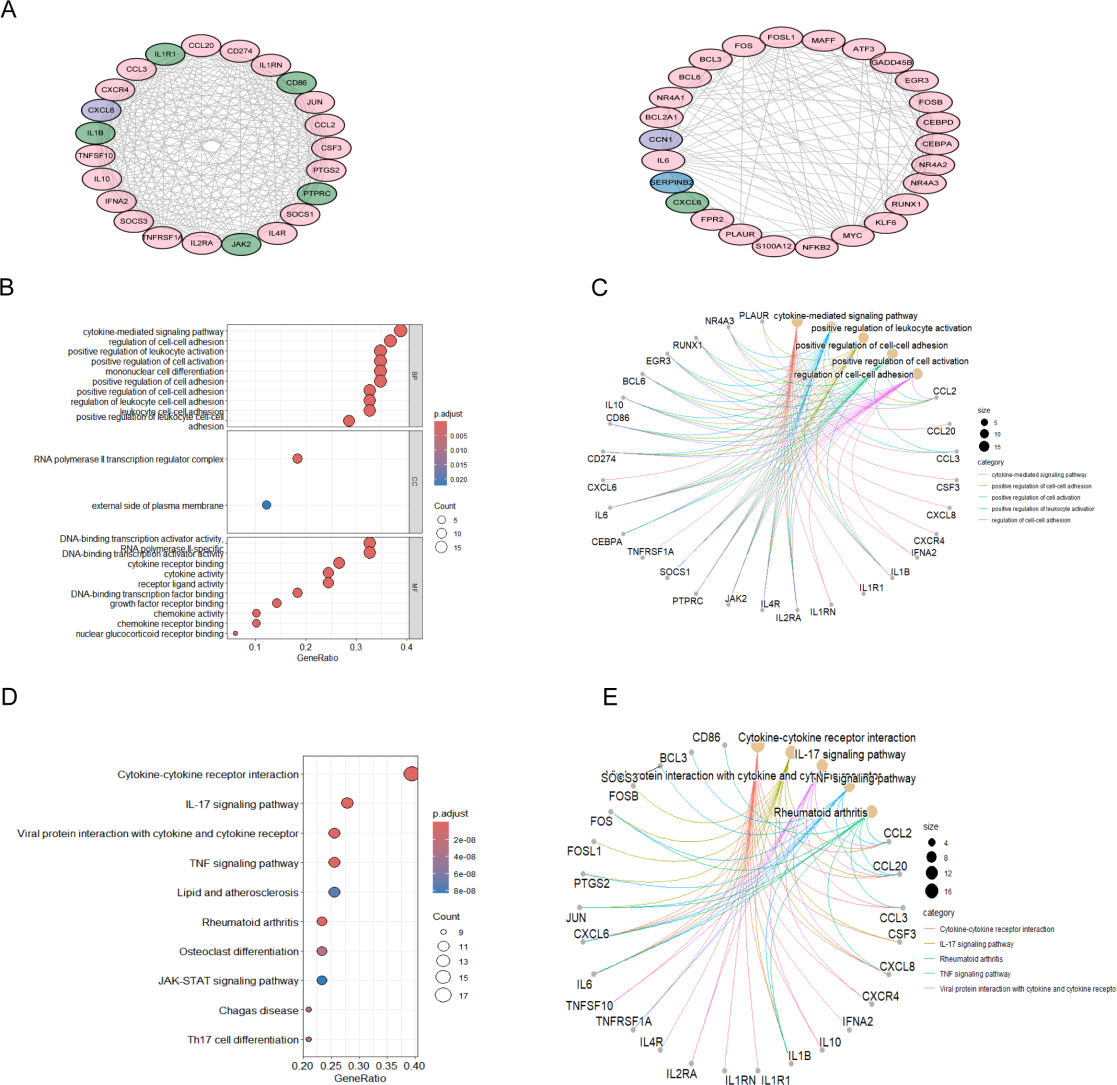
PPI analysis of T2DM-related secreted proteins and MAFLD key genes, followed by enrichment analysis. **(A)** PPI network of the top-scoring module 1 and module 2 genes analysed using the Cytoscape plug-in MCODE. Pink nodes represent key MAFLD genes, green nodes represent T2DM-related secreted proteins, and purple nodes represent genes common to both groups. **(B-E)** Analysis of the PPI network of module 2 genes with the highest scores based on GO and KEGG. The GO results of the genes included in modules A are displayed in bubble plots and circular plots, with similar plots constructed to illustrate the results of the KEGG analysis. PPI, protein-protein interactions; MCODE, molecular complex detection; GO, Gene Ontology; KEGG, Kyoto Encyclopedia of Genes and Genomes; BP, biological processes; CC, cellular component; MF, molecular function.

Using the STRING database as a basis, we examined 91 T2DM-associated secreted proteins that interacted with MAFLD genes, selecting those with median confidence scores greater than 0.4 to identify causal genes and underlying processes in T2DM-associated MAFLD. Cytoscape software visualized these causal genes, and MCODE was then employed to identify the top two most significant modules, from which 49 genes were found to be T2DM-associated pathogenic genes (**Figure 3A**). Next, we conducted GO enrichment and KEGG enrichment analyses of the relevant pathogenic genes to gain a deeper understanding of their roles and unique mechanisms. These genes were found to be abundant in cytokine-mediated signaling pathways, cell-cell adhesion regulation, and positive modulation of leukocyte activation, according to GO enrichment analysis. The KEGG pathway analyses indicated that the pathogenic genes in T2DM-associated MAFLD interacted with cytokine–cytokine receptor interactions and were closely associated with the IL-17, JAK-STAT, and TNF signaling pathways, among others (**Figures 3B–E**).

### 3.4 Screening of pivotal genes with diagnostic value through machine learning and cytoHubba

**Figure 4:**
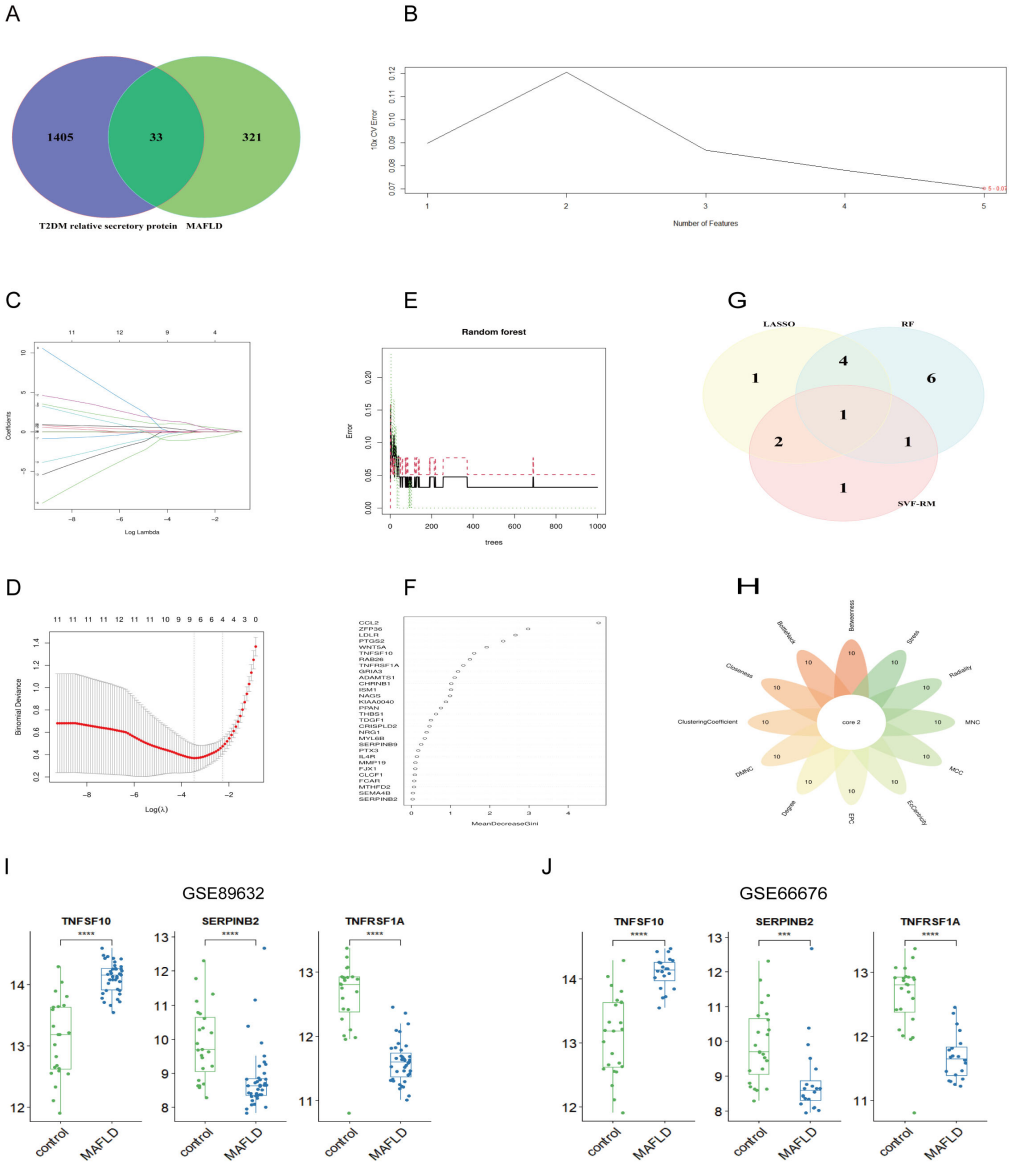
Machine learning combined with 12 algorithms in the cytoHubba plug-in to screen central genes. **(A)** The Venn diagram shows the MAFLD genes associated with T2DM secreted proteins; **(B)** SVM-RFE algorithm; **(C, D)** LASSO regression algorithm; **(E, F)** RF algorithms; **(G)** Venn diagram of three machine learning algorithms; **(H)** Venn diagram of 12 algorithms in the cytoHubba plug-in; **(I, J)** Expression of pivot genes in the internal GSE89632 datasets and external GSE66676 datasets. LASSO, minimum absolute shrinkage and selection operator; SVM-RFE, support vector machine-recursive feature elimination; RF, Random Forest. ***P < 0.001; ****P < 0.0001.

The analysis of T2DM-associated secretory proteins and important genes in metabolic associated fatty liver disease revealed 33 overlapping genes (**Figure 4A**). Using the SVM-RF machine learning algorithm, five genes were selected for further consideration in MAFLD (**Figure 4B**). Out of the 33 common genes, eight prospective candidate genes were identified as being able to accurately detect patients with T2DM-associated MAFLD (**Figures 4C, D**). The 33 common genes were sorted using an RF machine learning algorithm according to the variable importance of each gene. The genes with a mean-decreasing Gini score greater than 1 were extracted (**Figures 4E, F**). The only overlapping hub gene is TNFSF10 (**Figure 4G**), which was discovered by intersecting genes discovered using three machine learning methods. In a similar manner, we employed Cytoscape software and PPI network analysis on the 33 common genes to identify the causative genes in T2DM-associated MAFLD. Twelve algorithms in the CytoHubba plug-in identified two causative genes—SERPINB2 and TNFRSF1A (**Figure 4H**). In summary, TNFSF10, SERPINB2, and TNFRSF1A were identified as pathogenic genes related to MAFLD and T2DM. These three hub genes exhibit consistent patterns of expression in the internal (GSE89632) and external (GSE66676) datasets (**Figures 4I, J**).

### 3.5 Diagnostic utility of hub genes in MAFLD AND T2DM

**Figure 5:**
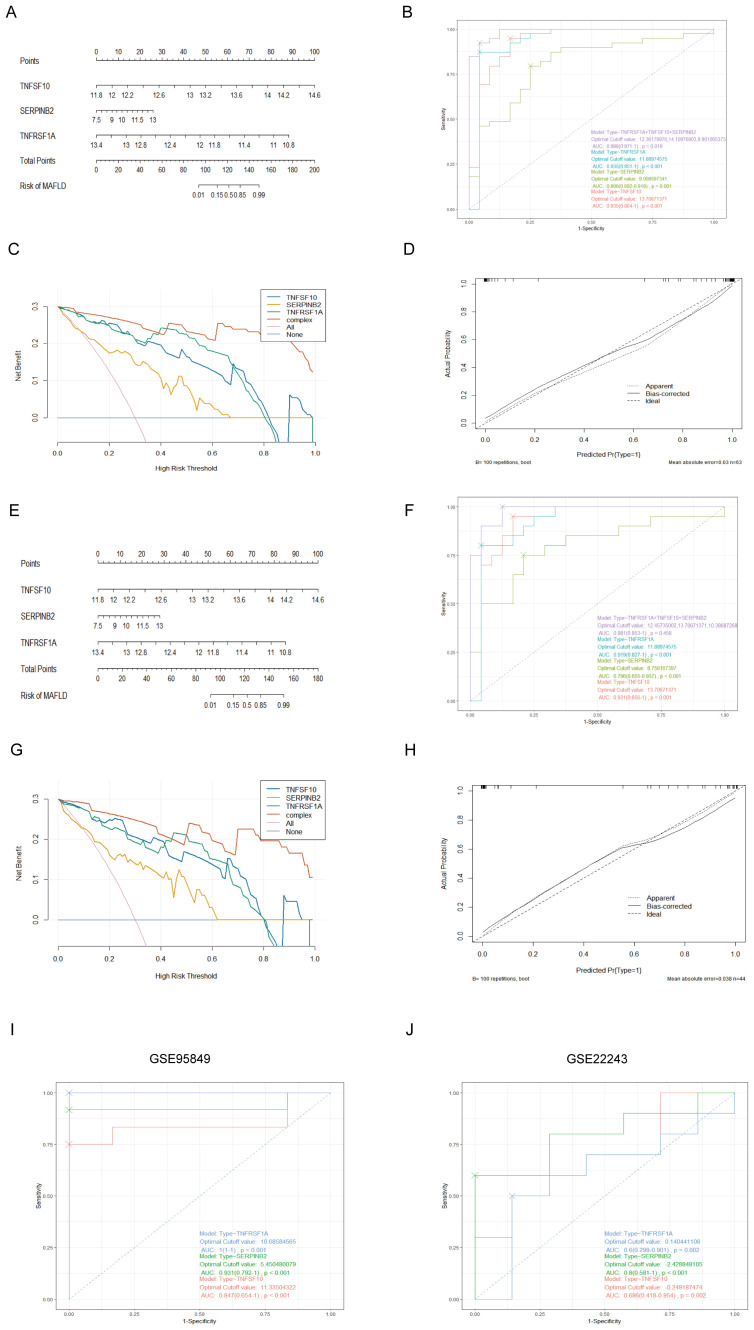
Establishment of the nomogram model and effect evaluation. **(A)** The nomogram suggested that the joint prediction of three hub genes was greater than that of a single hub gene. **(B)** The ROC curve showed that the nomogram had high diagnostic performance. **(C)** DCA decision curve indicates that intervention targeting all three hub genes is beneficial for MAFLD patients, with potential for enhanced benefit when all three genes are simultaneously addressed. **(D)** The calibration curves predicted by the nomogram model of T2DM-associated MAFLD suggest that the model is accurate. **(E-H)** Nomogram, ROC curve, DCA decision curve, calibration curves based on the constructed GSE66676. **(I, J)** The ROC curve of 3 T2DM-related hub genes in date set GSE95849, GSE22243.

We created an alignment diagram using logistic regression analysis to build a diagnostic model for MAFLD and investigate the diagnostic utility of the three hub genes (**Figure 5A**). The area under the curve (AUC) values for each hub gene and the alignment diagram were evaluated using an ROC curve to ascertain their sensitivity and specificity for the diagnostic efficacy of T2DM-associated MAFLD. The AUC values for all three hub genes exceeded 0.8, demonstrating strong predictive value. In addition, the alignment diagram’s AUC values were notably higher (**Figure 5B**). DCA suggested that the alignment diagram model could enhance decision-making in diagnosing T2DM-associated MAFLD (**Figure 5C**). Furthermore, the calibration curves showed that the projected probabilities of the ideal model closely matched those of the developed diagnostic model (**Figure 5D**). We obtained similar results in the validation dataset GSE66676 (**Figures 5E–H**). When considered collectively, these findings highlight the substantial diagnostic utility of the alignment diagram for MAFLD. Furthermore, in two datasets of T2DM, GSE95849 and GSE23343, the AUC values of the three hub genes (TNFSF10, SERPINB2, TNFRSF1A) were 0.847, 0.931, 1 and 0.686, 0.8, 0.6, respectively (**Figures 5I, J**). Therefore, these three MAFLD-related genes may serve as effective markers for the diagnosis of type 2 diabetes, and they may play an important role in the development of comorbid T2DM in patients with MAFLD.

### 3.6 Analysis of hub genes and immune cell infiltration in MAFLD

**Figure 6:**
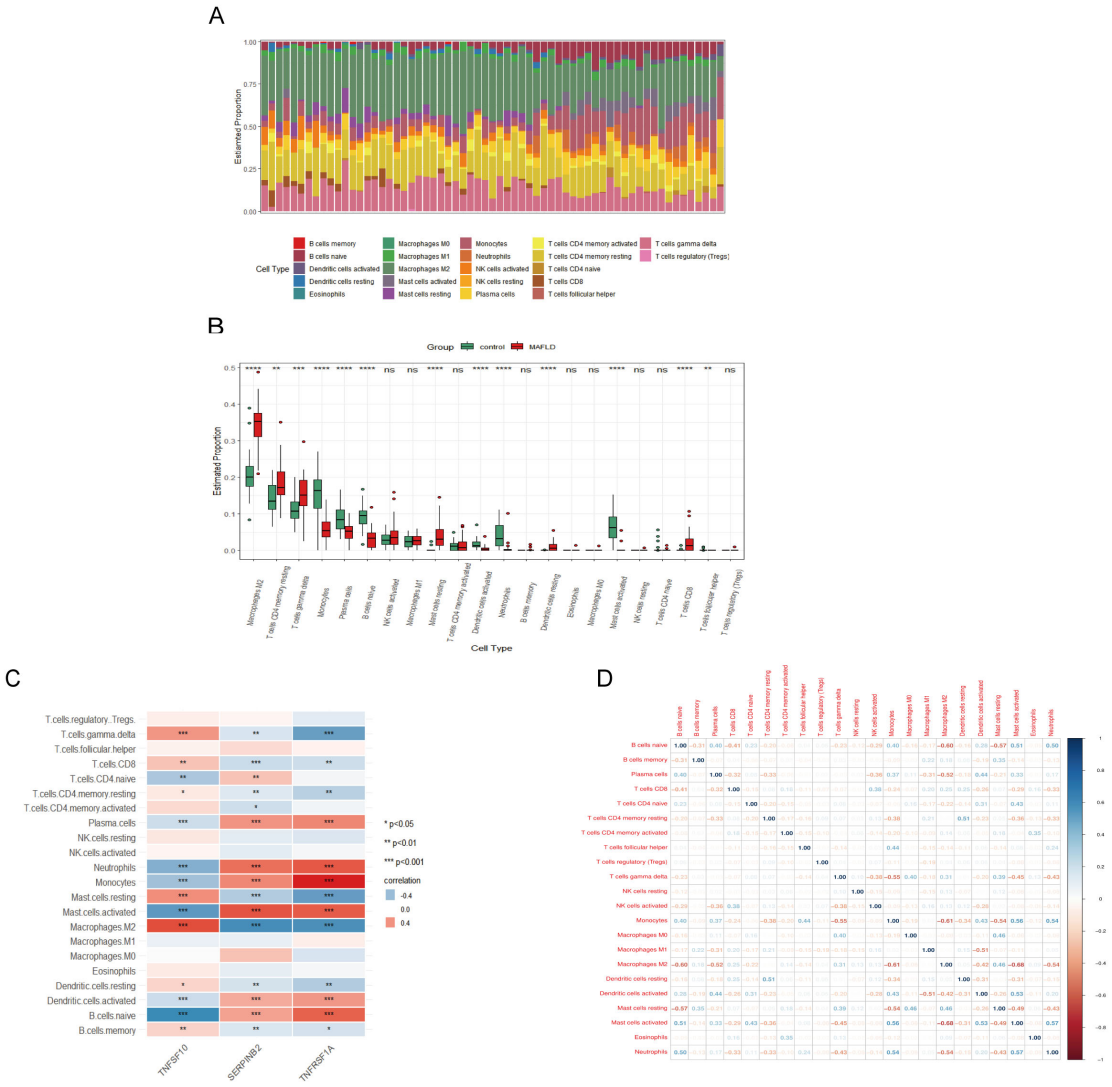
MAFLD immune cell infiltration assay. **(A)** Histogram showing the proportion of immune cells between the MAFLD group and the control group. **(B)** Box plot comparing 22 immune cells between the MAFLD and control groups. **(C)** Correlation map showing the relationship between the differentially infiltrating immune cells and the three hub genes. **(D)** Heat map depicting the correlation of infiltration levels among the 22 immune cells. *P < 0.05; **P < 0.01; ***P < 0.001; ****P < 0.0001; ns, not significant.

Using the CIBERSORT algorithm, we determined the immune cell features that were significantly different across 13 immune cell subpopulations between liver samples from the fatty liver group and the control group, providing insights into immunological infiltration in MAFLD (**Figures 6A, B**). We found that all three hub genes were significantly correlated with the presence of immune cells in MAFLD (**Figure 6C**). In addition, a correlation analysis of the 22 immune cells revealed a significant positive correlation between monocytes and mast cell activation, as well as a negative correlation between mast cell activation and M2 macrophages (**Figure 6D**).

### 3.7 Hub gene prediction transcription factors and miRNAs in MAFLD

**Figure 7:**
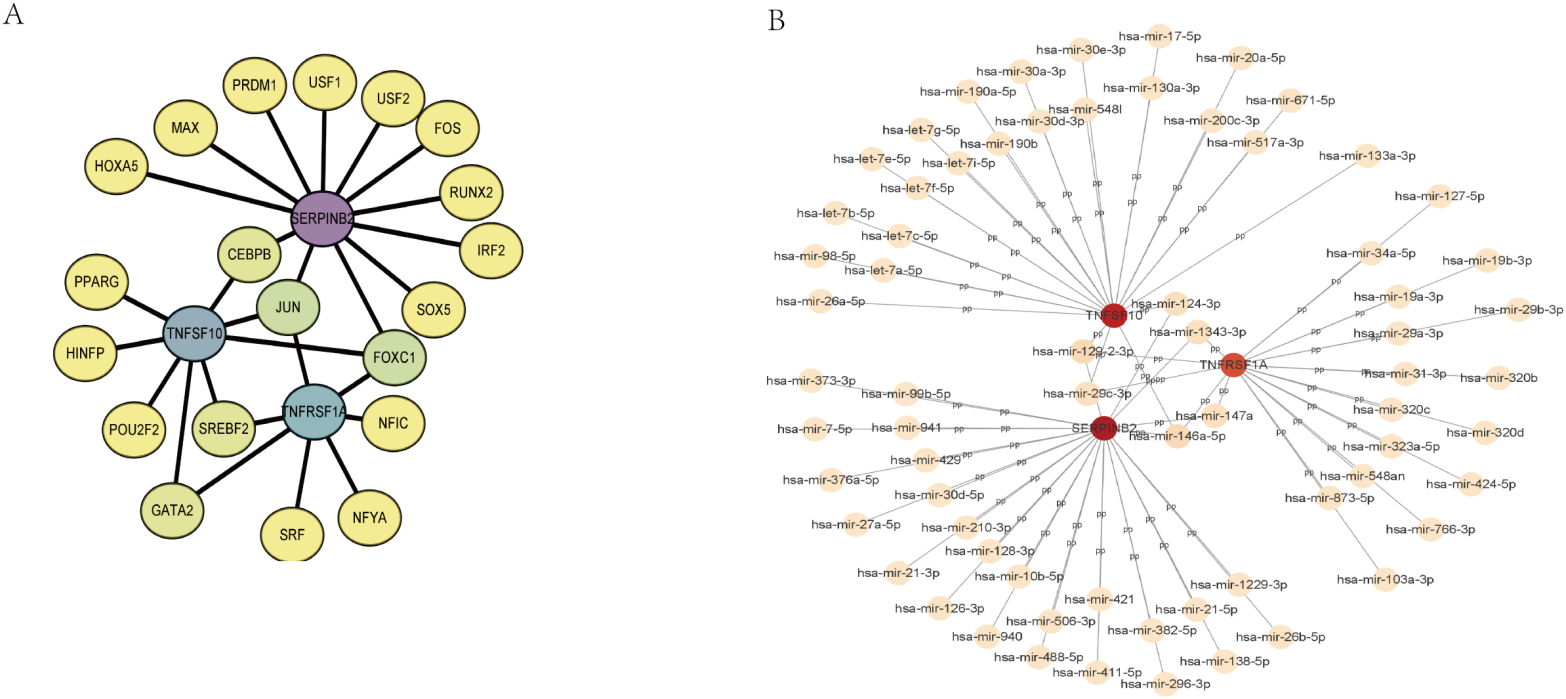
Regulatory network. **(A)** TF-gene interaction network of hub genes. **(B)** The network of miRNAs interacting with hub genes. TF, transcription factor; miRNA, microRNA.

To uncover the possible molecular mechanisms of SERPINB2, TNFSF10, and TNFRSF1A in T2DM combined with MAFLD disease, we analyzed the interactions between these three genes and transcription factors. We used the NetworkAnalyst website to access the JASPAR database in order to predict transcription factors (TFs) (**Figure 7A**), of which eight had degrees greater than two: SERPINB2, TNFSF10, TNFRSF1A, FOXC1, JUN, CEBPB, GATA2 and SREBF2. We also use the miRTarBase database to predict potential upstream miRNAs for TNFRSF1A, TNFSF10, and SERPINB2. Finally, we used Cytoscape software to visualize the results, showing that six miRNA degrees were greater than two (**Figure 7B**).

### 3.8 Expression of TNFSF10, SERPINB2, and TNFRSF1A at blood and the cellular level

**Figure 8:**
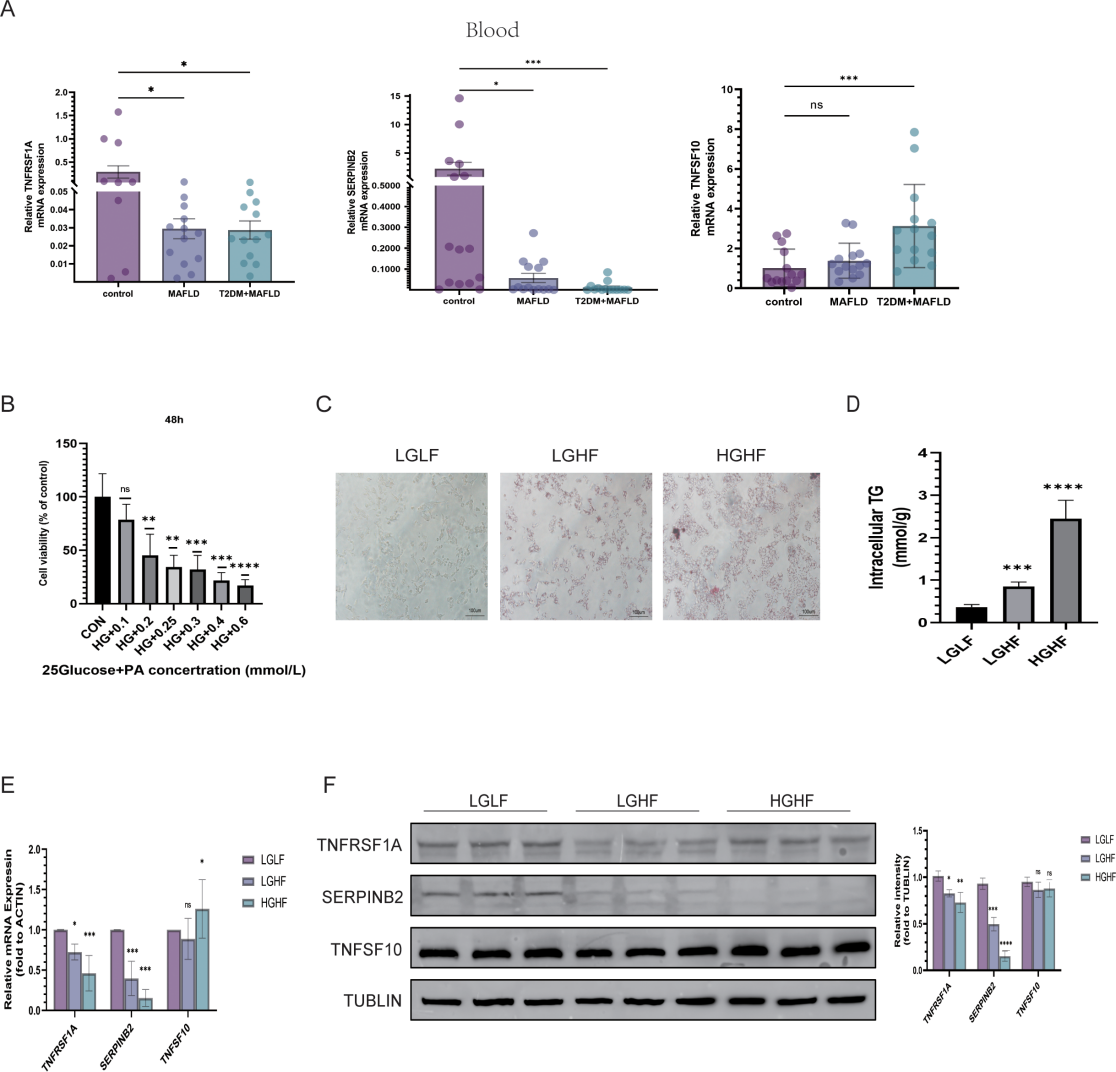
Validation of three hub gene expression patterns. **(A)** Expression of TNFSF10, SERPINB2, and TNFRSF1A in the blood of healthy people, MAFLD patients, people with type 2 diabetes and MAFLD; **(B)** Viability of HepG2 cells treated with different concentrations of PA for 48 h; **(C)** Oil Red O staining of HepG2 cells treated with LGLF, LGHF, and HGHF for 48 h (magnification × 200, scale 100 µm); **(D)** TG content of HepG2 cells after 48 h of HG and PA treatment; **(E, F)** RT-qPCR/WB was used to detect the expressions of *TNFSF10*, *SERPINB2*, and *TNFRSF1A*. PA, sodium palmitate; HG, represents the high-glucose group; HF, represents the high-fat group; (*P < 0.05, **P < 0.01, ***P < 0.001, ****P < 0.0001; ns, not significant).

Subsequently, Quantitative real-time reverse transcription polymerase chain reaction (qRT-PCR) was used to analyze the expression of TNFSF10, SERPINB2, and TNFRSF1A in blood samples from patients with normal, MAFLD, and T2DM-combined MAFLD. The results of the study showed that while TNFSF10 expression was significantly higher in patients with T2DM and MAFLD, the expression levels of TNFRSF1A and SERPINB2 in the blood of patients with MAFLD and T2DM combined with MAFLD were significantly lower compared to healthy individuals (P<0.05) (**Figure 8A**).

At the same time, we mixed PA with different glucose concentrations to mimic the high glucose and high fatty acid environment to better study the expression of 3 genes in MAFLD and T2DM combined with MAFLD. We first determined that sugar concentrations of 5.5 and 25 mmol/L simulated the normal and diabetic environments of the body, respectively. On this basis, we combined PA to simulate the high fatty acid environment and established the cell models of the LGHF group and HGHF group in order to further investigate the expression of TNFSF10, SERPINB2, and TNFRSF1A in MAFLD and T2DM-related MAFLD. We used the CCK-8 assay to measure the PA concentration, which was determined to be 200 µmol/L. Oil Red O staining results revealed that lipid accumulation in HepG2 cells in the LGHF and HGHF groups increased gradually over time compared to the control group. These results showed that the combined fatty liver and diabetes models were successfully developed, and the results of the quantitative analysis of triglycerides were consistent with the preceding findings (**Figures 8B–D**).

The protein expression levels of TNFSF10, SERPINB2, and TNFRSF1A were measured in relation to various glycolipid concentrations using qRT-PCR and Western Blotting (WB). The findings demonstrated that, after lipid overload, the expression of TNFRSF1A and SERPINB2 decreased in the low- glucose and high-glucose groups relative to the low-glucose and low-fat (LGLF) group (P < 0.05). Additionally, there was a tendency for the expression of both to be lower in the high-glucose and high-fat environment than in the high-fat group alone. Nevertheless, TNFSF10 expression levels in HepG2 cell lines did not significantly alter before and after the fat-only treatment or the high glucose plus high-fat treatment, as assessed using both transcriptional and protein levels (**Figures 8E, F**).

## 4 Discussion

In order to investigate new genes, potential diagnostic or prognostic biomarkers, underlying mechanisms, and possible therapeutic targets based on big data, microarray and sequencing methods, integrated bioinformatics analysis, and machine learning tools have increasingly been applied in recent years (35–37). This has facilitated the investigation of causative factors and potential mechanisms of complex diseases. Therefore, in order to treat T2DM-associated MAFLD, we employ a range of integrated bioinformatics analytic approaches along with machine learning to identify the causal genes of T2DM-associated MAFLD in order to identify novel therapeutic targets by investigating the critical genes and their mechanisms. This will enable us to intervene early in the disease’s progression and halt its advancement. To uncover three T2DM-associated MAFLD-centred genes, we combined machine learning, WGCNA, and PPI networks with transcriptome datasets obtained from the GEO database. Following modelling for prognosis and diagnosis, validation of predictive capacity, and biological studies, we determined that SERPINB2 and TNFRSF1A are frequent diagnostic markers of MAFLD, exhibiting consistent expression patterns in MAFLD and T2DM-associated MAFLD, and are strongly linked to the onset of T2DM.

As a component of the TNFSF/TNFRSF system, TNFRSF1A primarily encodes the TNFR1 protein, which is one of the main TNFα receptors. In addition to its pro-inflammatory characteristics, the activation of this gene can lead to apoptosis and other types of cell death (38). Furthermore, there is mounting evidence linking TNFRSF1A to hepatic glucolipid metabolic abnormalities. Reports indicate that in mutant mice with sustained cell surface expression of the TNFRSF1A receptor, this constitutive TNFR1 signaling exacerbates the pro-inflammatory and pro-fibrotic features of non-alcoholic steatohepatopathy but is not linked to the appearance of hepatic steatosis (39). Similarly, after the targeted reduction of TNFR1 in hepatocytes from mice fed a Western fast-food diet, insulin resistance and glucose tolerance were markedly reduced (40). In particular, Lambertucci et al. established a MAFLD model using TNFR1 systemic knockout mice and found that the disruption of TNFR1 signaling increased plasma IL-1β levels and intensified the hepatic inflammatory response, insulin resistance, and liver injury. This suggests that the disruption of TNFR1 signaling accelerates the progression of hepatic conditions from simple steatosis to a more severe phenotype exhibiting many features of non-alcoholic steatohepatitis (41). Therefore, when considered collectively, the significance of TNFRSF1A expression in MAFLD is debatable. According to our research, the liver tissues of individuals with MAFLD in the database exhibited lower levels of TNFRSF1A expression, which correlated with higher diagnostic accuracy values (AUC > 0.80). The same results were confirmed in the patient’s blood and a cellular model where a high-fat environment caused a decline in TNFRSF1A expression, with the downregulation being more pronounced in a high-glucose and high-fat environment. Overall, according to our findings, TNFRSF1A could serve as a diagnostic marker for both MAFLD and MAFLD linked to T2DM.

In this study, SERPINB2 was identified as a significant marker for the diagnosis of MAFLD patients with T2DM. SERPINB2 belongs to the human serine protease inhibitor (SERPIN) superfamily, which is mainly expressed in activated monocytes, macrophages, fibroblasts, and endothelial cells. It is primarily found as an intracellular non-glycosylated protein as well as a secreted glycosylated protein (42, 43). SERPINB2 is significantly expressed in activated monocytes, macrophages, fibroblasts, and endothelial cells and is primarily implicated in the connections between cellular inflammation, senescence and maladaptive repair (44–46). SERPINB2 has been implicated in many exogenous inflammatory and autoimmune diseases; however, to date, there is relatively little research on its role in liver disease (47, 48). In one study, Carson et al. utilized RNA sequencing to quantitatively analyze transcriptome changes in LX-2 cells, an activated human hepatic stellate cell line, before and after TGF-b1 induction and found that SERPINB2 was one of the most significantly dysregulated genes. Notably, although it currently has no characteristic role in hepatic stellate cell activation or fibrosis, this suggests that SERPINB2 may be a new marker for the identification of liver fibrosis (49). Furthermore, Silvia et al. discovered a significant increase in the expression of SERPINB2 in MAFLD compared to simple steatosis, based on gene expression experiments in liver tissues from patients with non-alcoholic steatohepatitis. This finding indicates that SERPINB2 may be associated with the inflammatory progression of MAFLD (50). In addition, through multiple bioinformatics analyses, we found that this gene may be a common pathogenic factor in the pathogenesis of T2DM and MAFLD. In line with the bioassay findings, our experimental results also demonstrated a significant drop in SERPINB2 expression levels in blood and high-fat and high-glucose cell modles.

Furthermore, we investigated immune cells exhibiting similar trends of immune microenvironment alterations in MAFLD. Our findings revealed that the core genes TNFRSF1A and SERPINB2 were strongly correlated with immune cell infiltration, suggesting that these potential biomarkers may not only distinguish MAFLD but also contribute to MAFLD by interacting with the inflammatory immune pathway. Based on the important roles of FOXC family (51), JUN (52, 53), and C/EBPβ (54) in glycolipid metabolism, we predicted that TNFRSF1A and SERPINB2 may play an important role in the pathogenesis of MAFLD and T2DM combined with MAFLD by regulating FOXC1, JUN, and C/EBPβ transcription factors. Similarly, mir-129-2-3p, mir-146a-5p, mir-124-3p, mir-29c-3p, mir-1343-3p, and mir-147a may be upstream miRNAs for TNFRSF1A and SERPINB2. This provides relevant insights into the function and mechanisms of disease-causing genes.

Our study has limitations despite the validation of results using various techniques. For instance, the exact mechanisms by which TNFRSF1A and SERPINB2 regulate the development of T2DM-associated MAFLD remain unclear and require further research. We have successfully identified two pathogenic genes associated with T2DM-related MAFLD, a discovery that offers a fresh perspective for delving into the pathological roles of these two genes in T2DM-related MAFLD.

## 5 Conclusion

Our research revealed that TNFRSF1A and SERPINB2 have the potential to be causative factors and diagnostic markers in T2DM-associated MAFLD. This finding is crucial for further studying the pathogenesis of T2DM-MAFLD and understanding the pathophysiology of both conditions. In conclusion, our research may open new opportunities for personalized, precision diagnostics and prevention for patients with T2DM-related MAFLD.

## Data Availability

The datasets presented in this study can be found in online repositories. The names of the repository/repositories and accession number(s) can be found in the article/**Supplementary Material**.
