# Supplementary figures and images for: Identification of biomarkers for the diagnosis of type 2 diabetes mellitus with metabolic associated fatty liver disease by bioinformatics analysis and experimental validation
Source: Front Endocrinol (Lausanne). 2025 Jan 28;16:1512503. doi: 10.3389/fendo.2025.1512503 (PMC11810736; doi:10.3389/fendo.2025.1512503)

Oil red O staining---LGLF

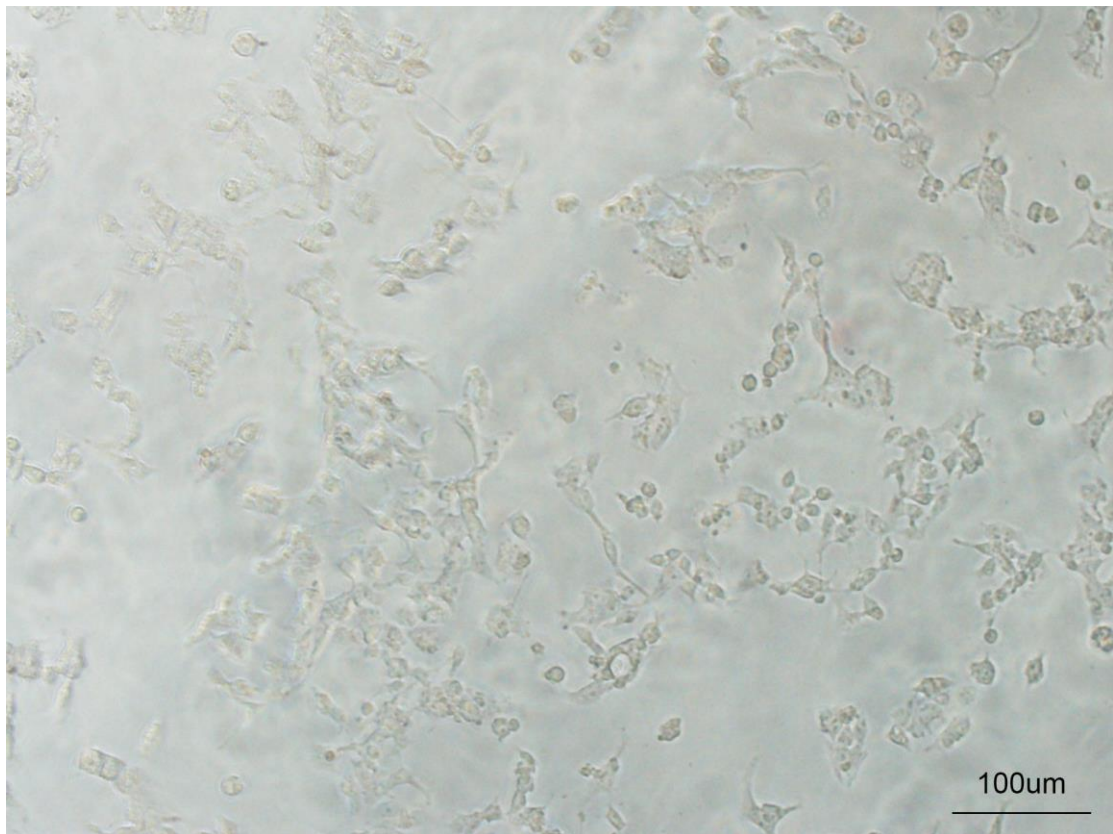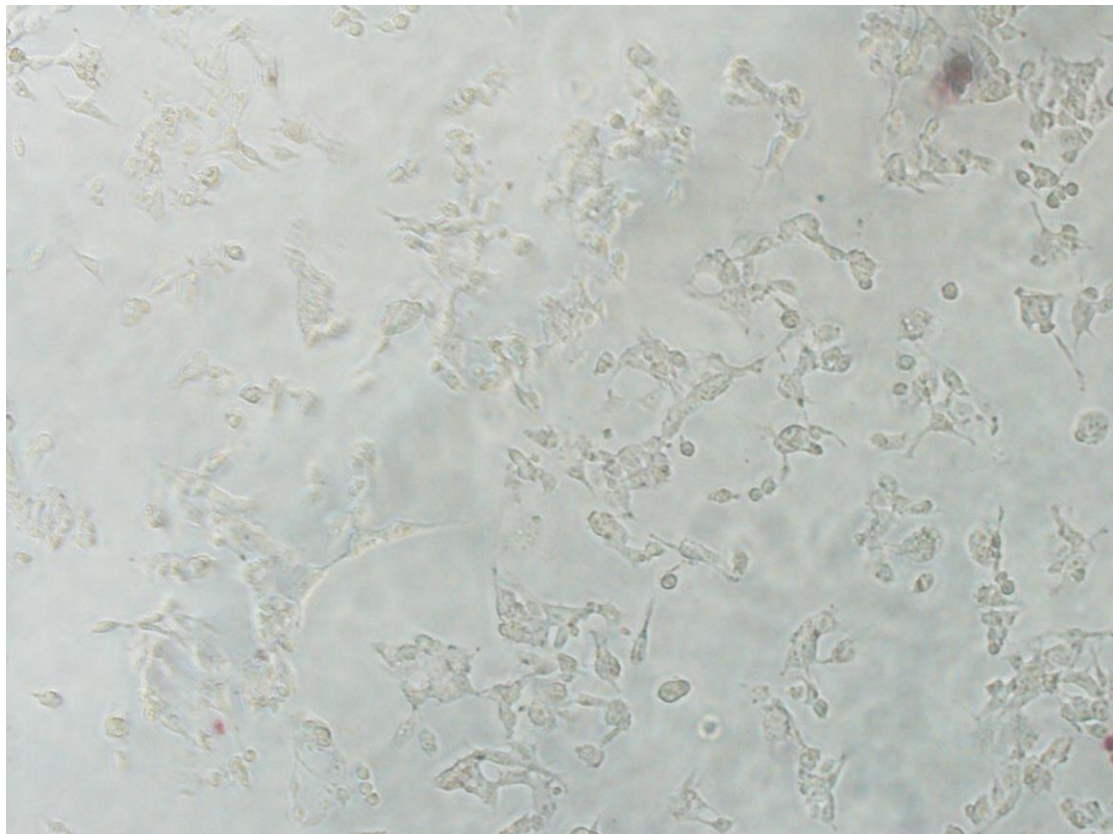

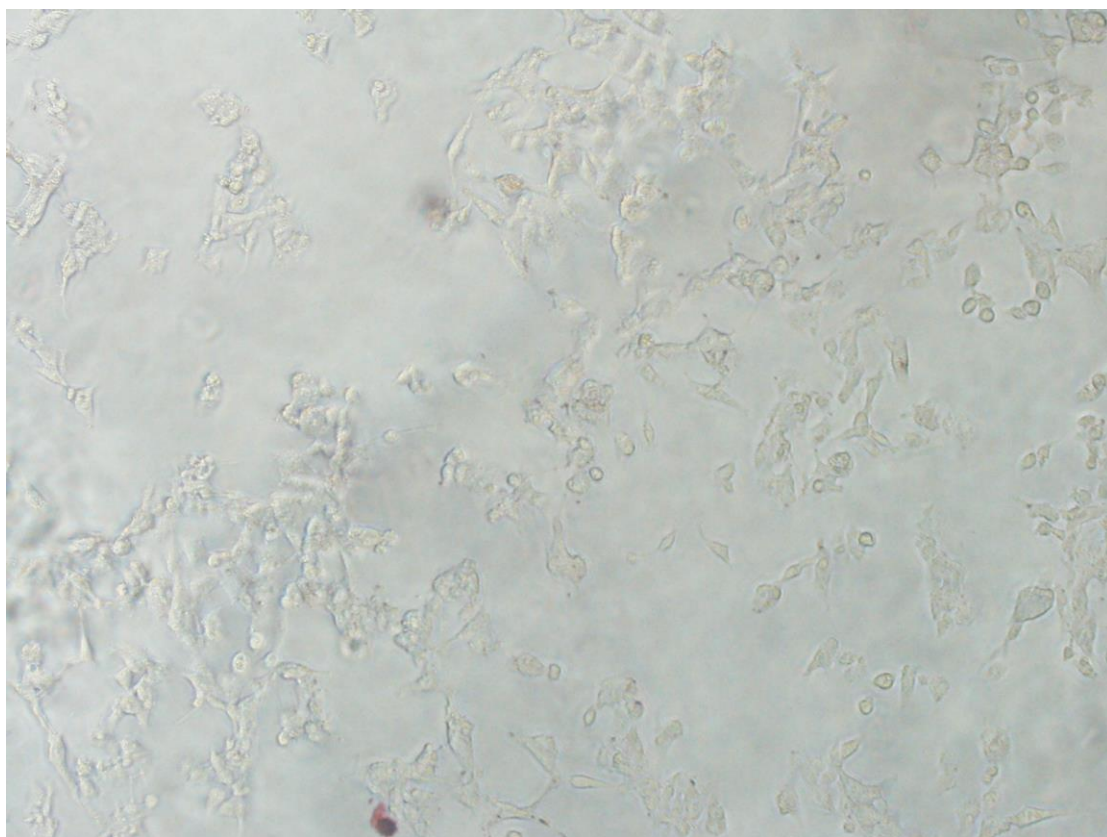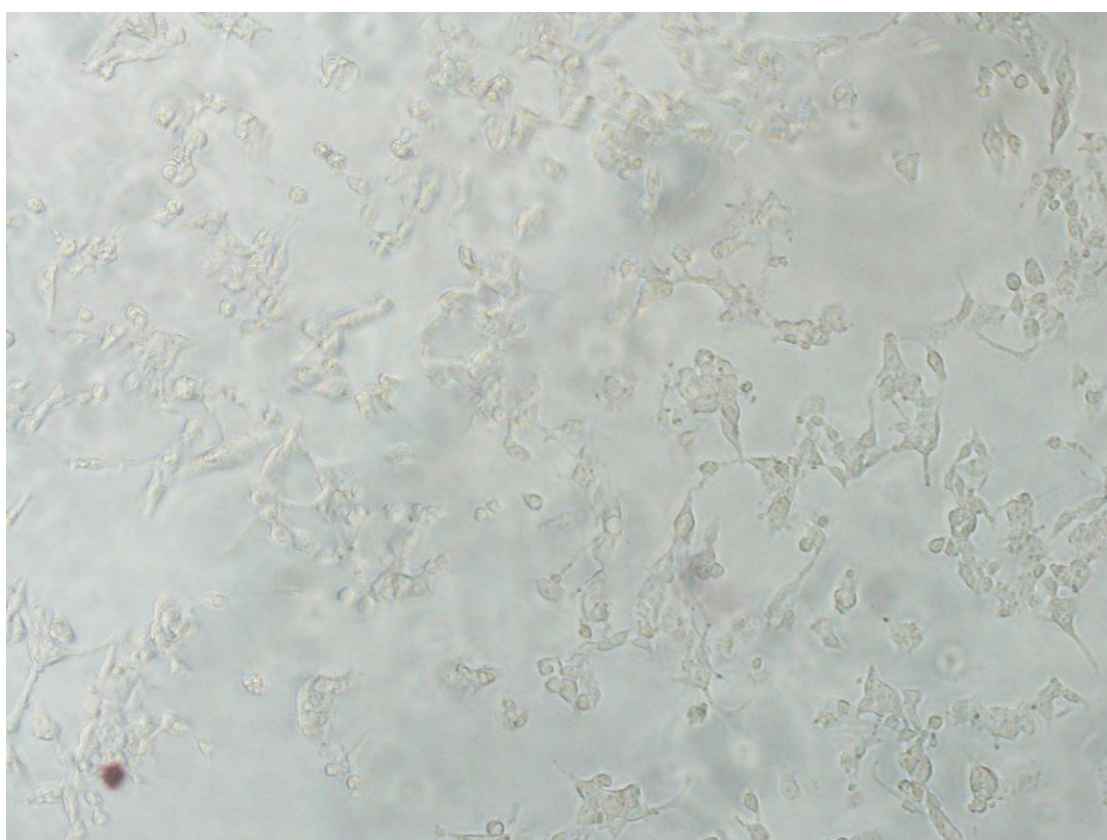

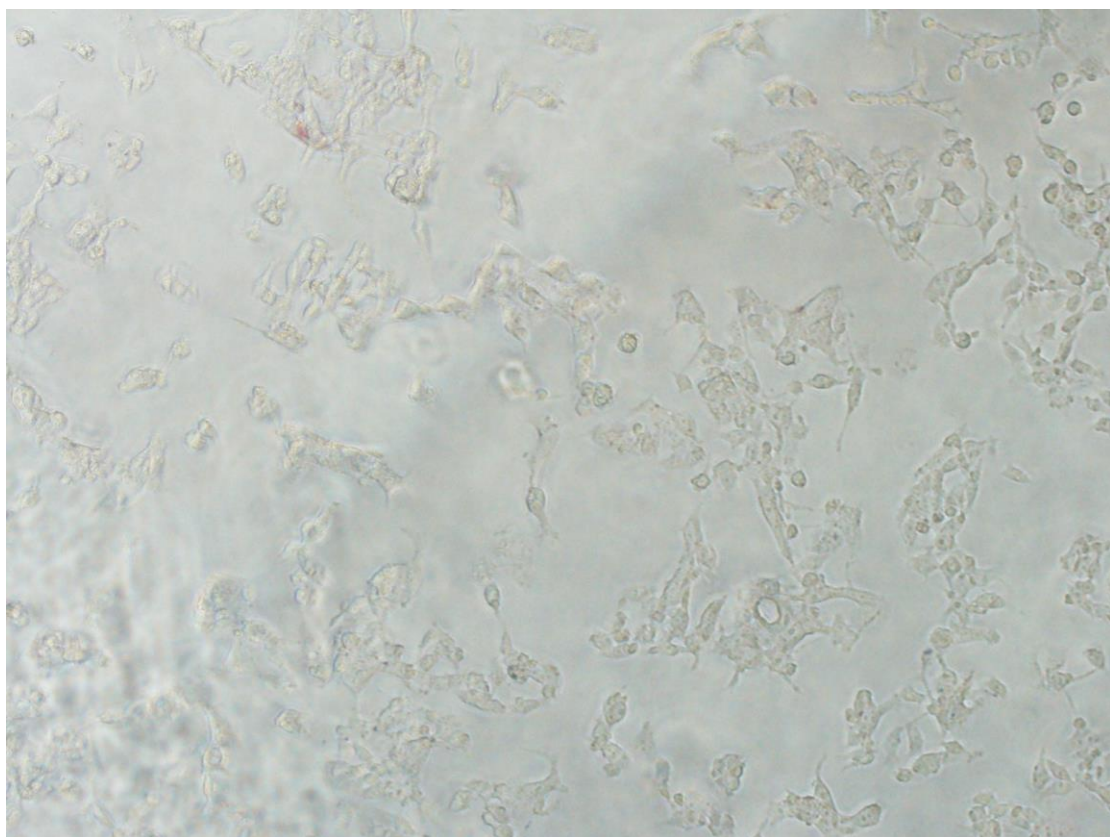

Oil red O staining—LGHF

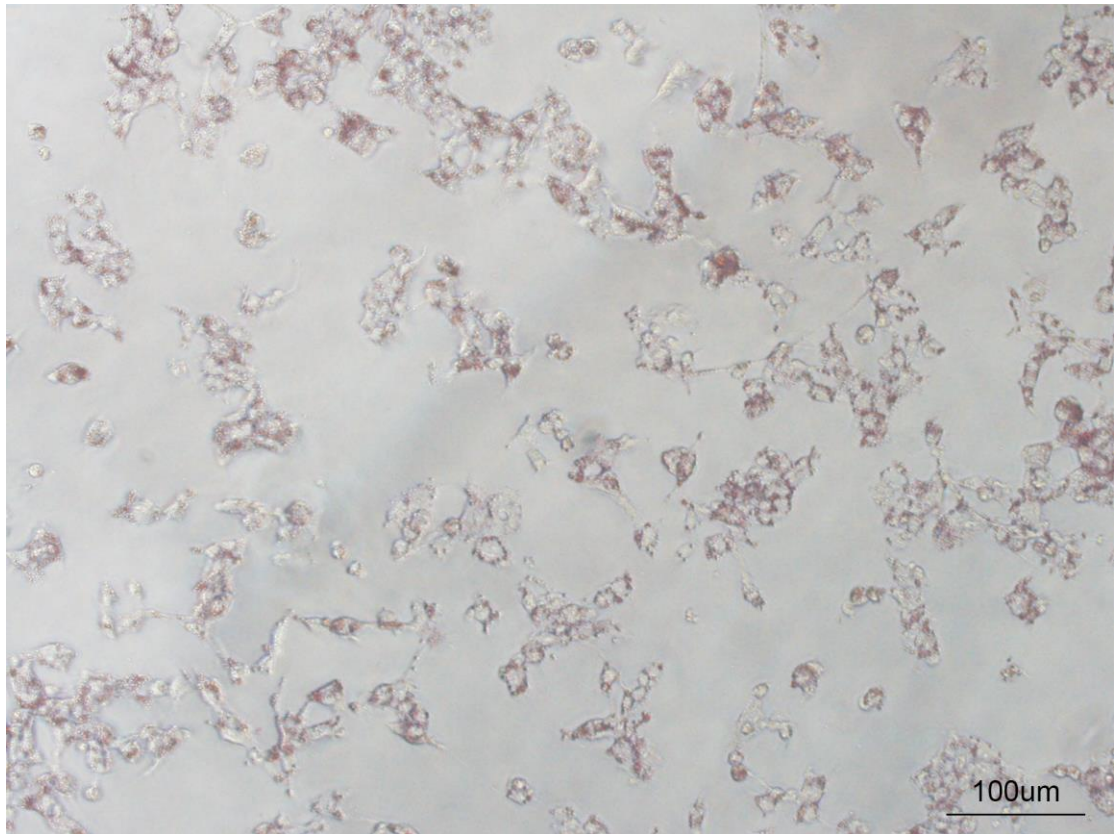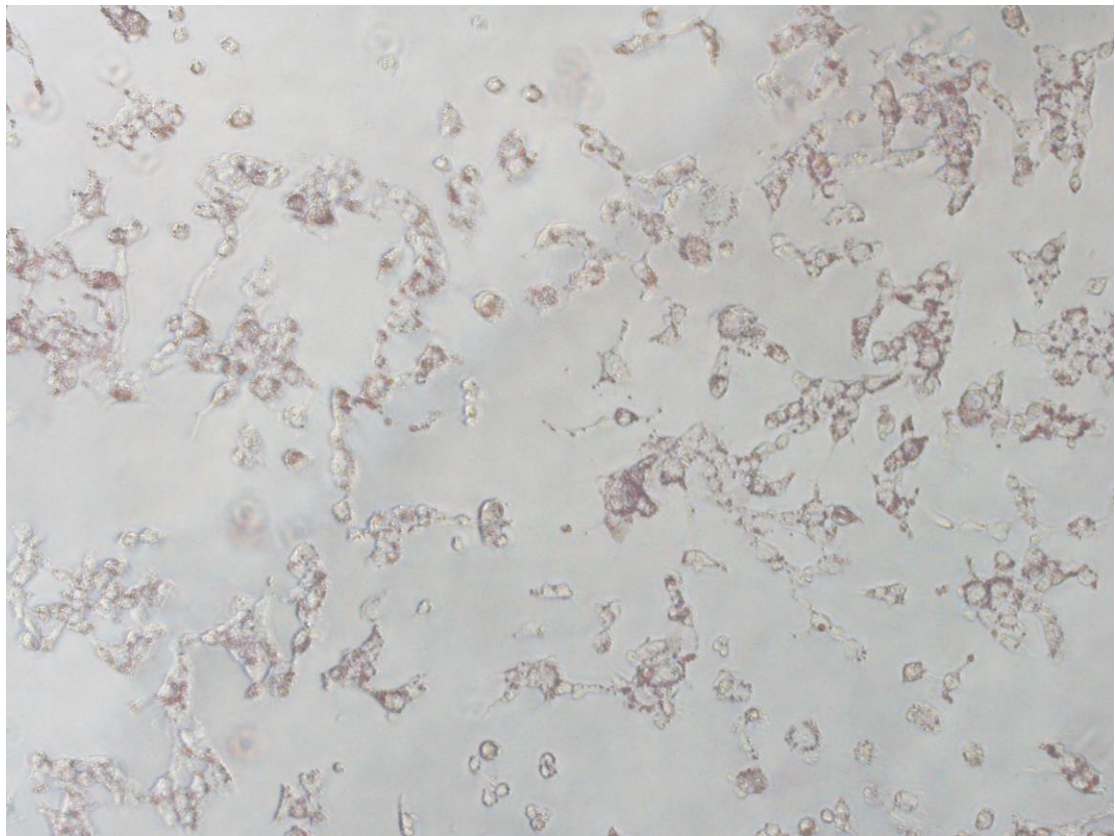

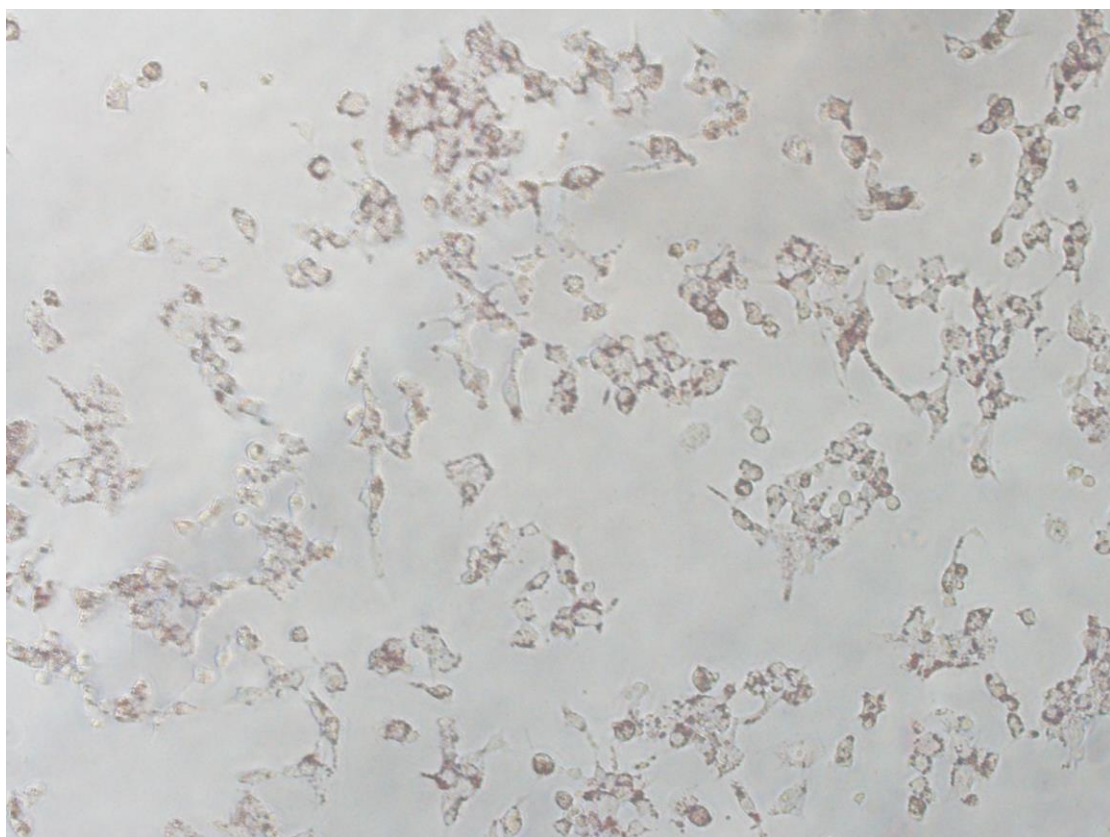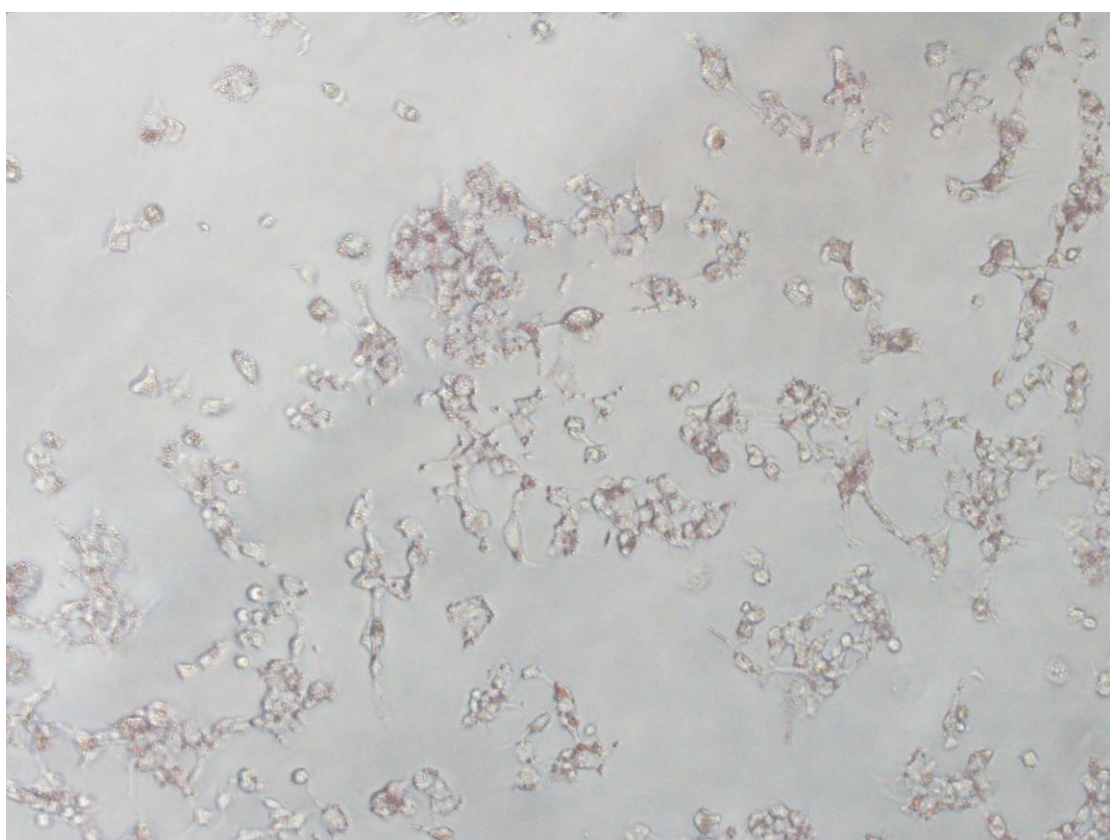

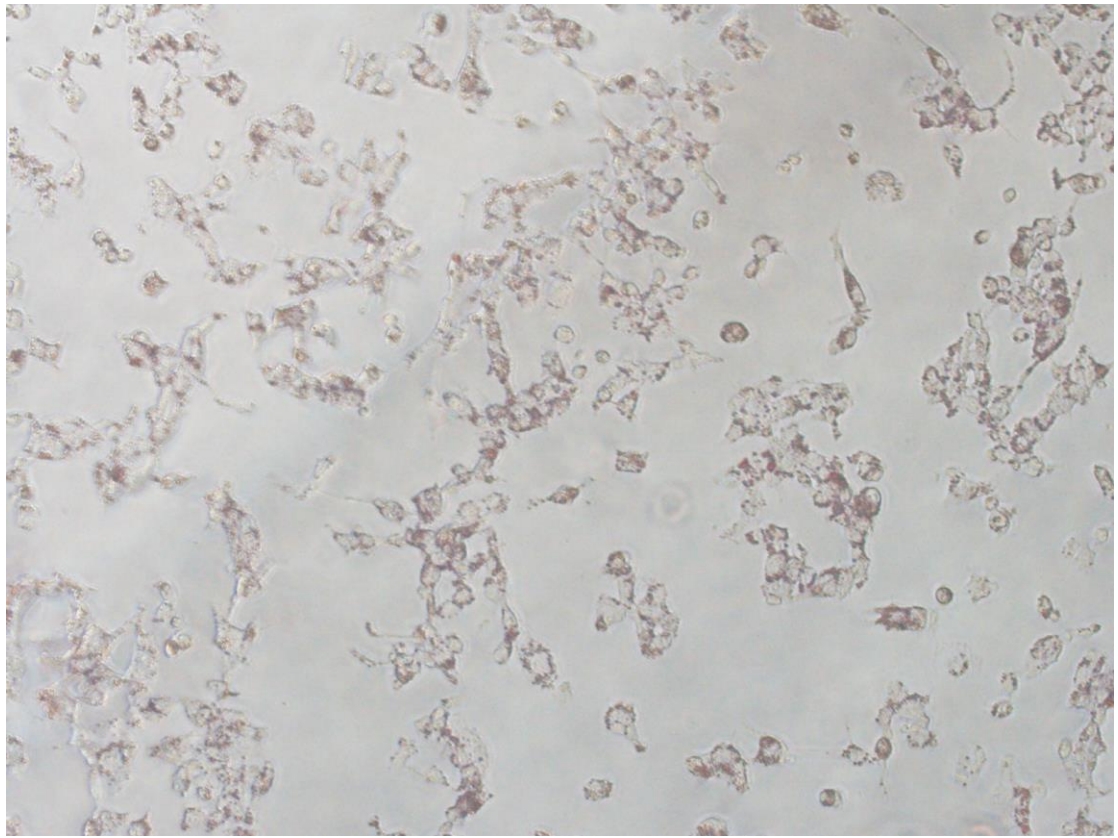

Oil red O staining—HGHF

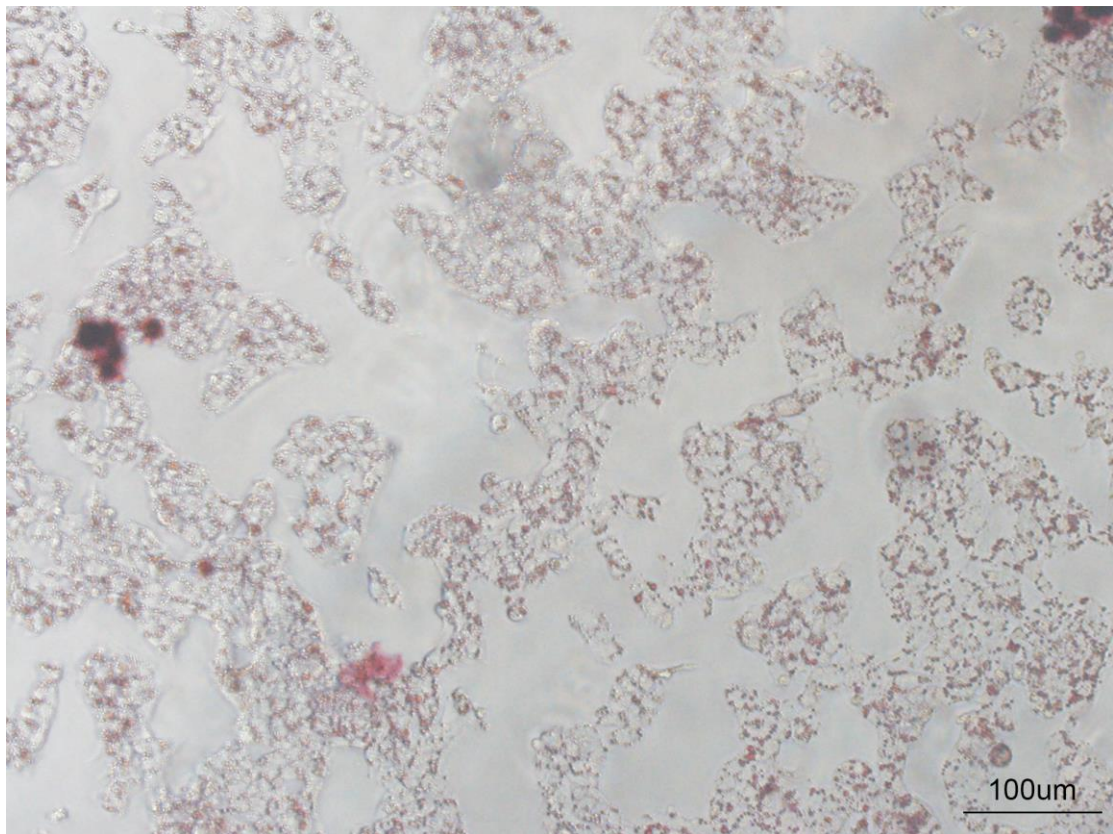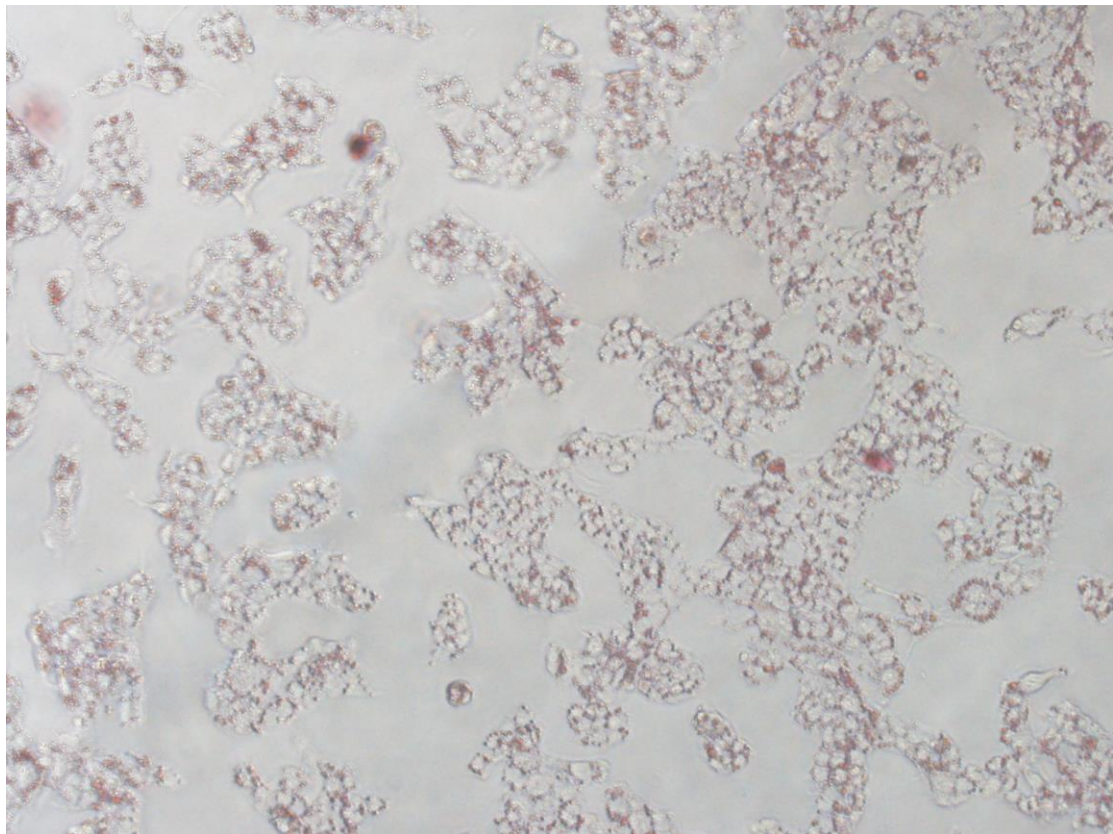

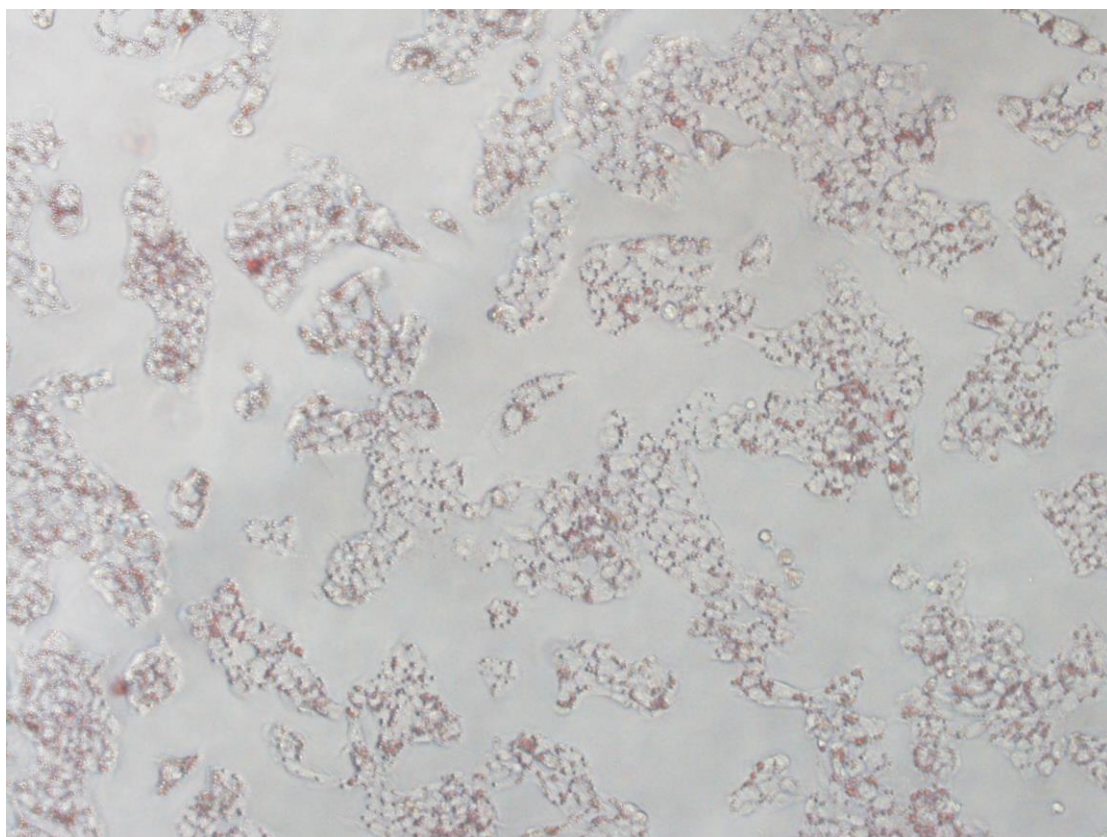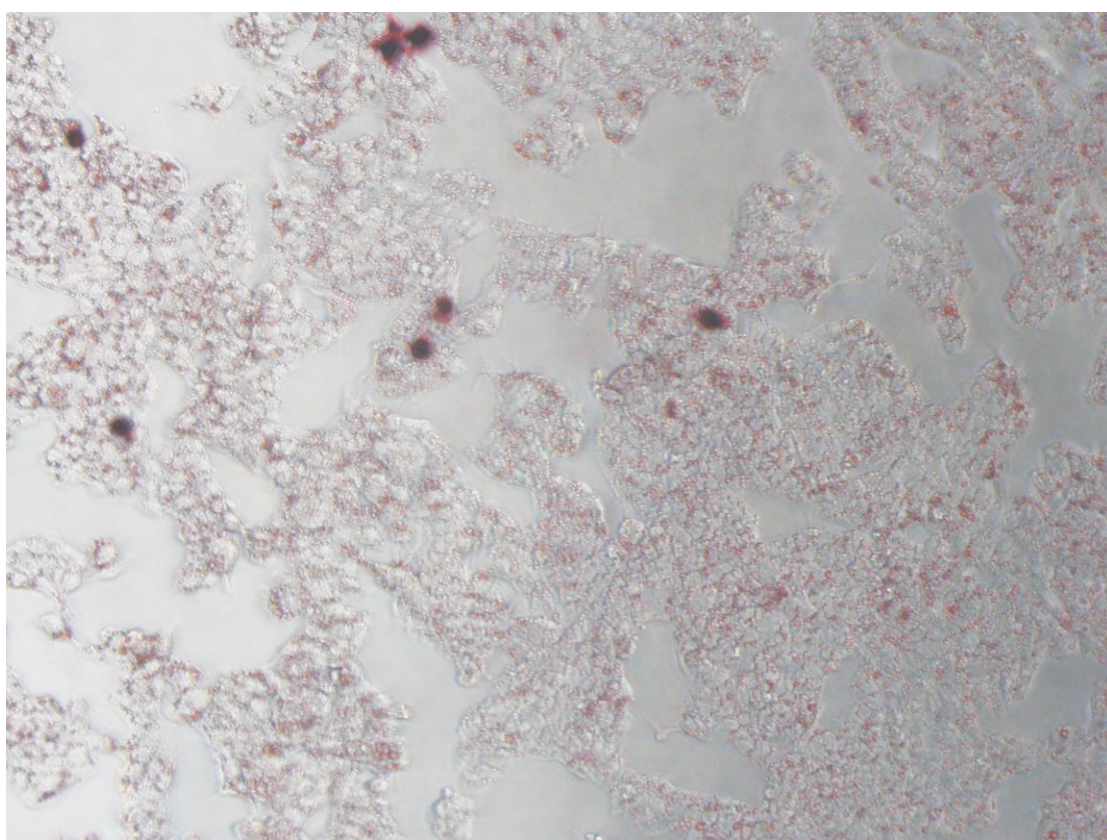

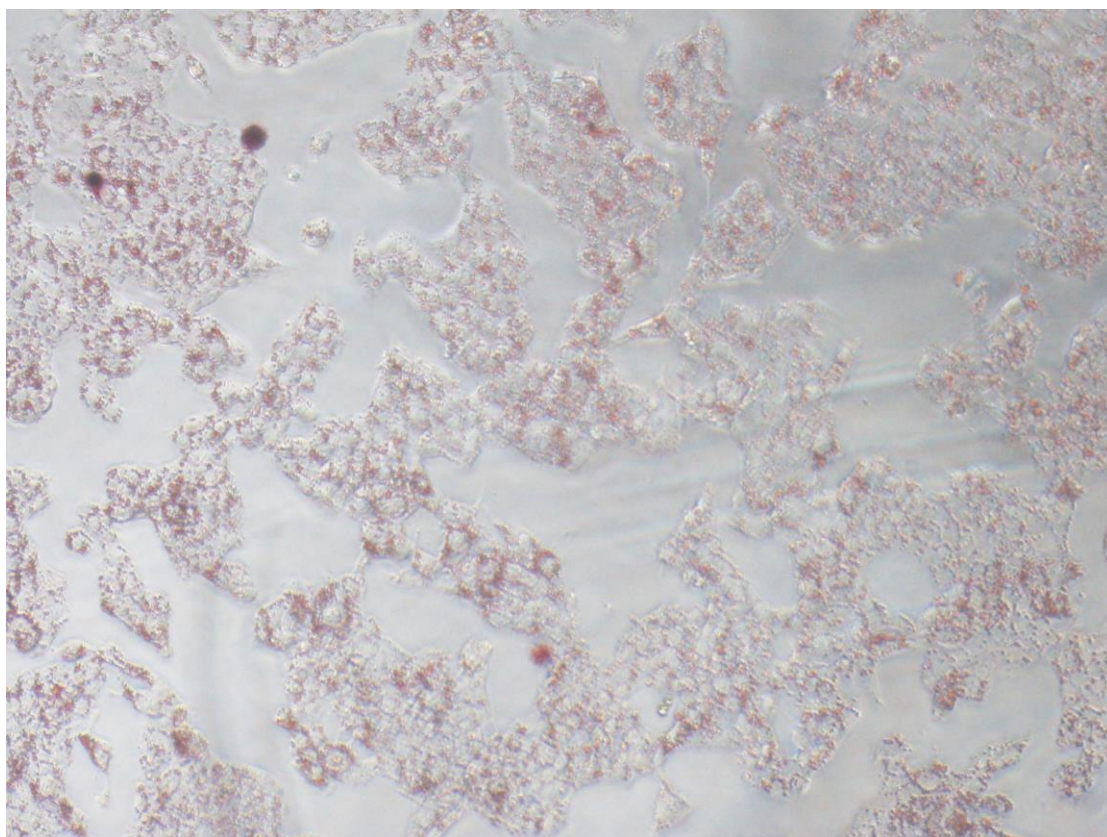

Supplement: Supplementary file 1 [file DataSheet1.pdf]
