# Supplementary material for: Identification of biomarkers for the diagnosis of type 2 diabetes mellitus with metabolic associated fatty liver disease by bioinformatics analysis and experimental validation
Source: Front Endocrinol (Lausanne). 2025 Jan 28;16:1512503. doi: 10.3389/fendo.2025.1512503 (PMC11810736; doi:10.3389/fendo.2025.1512503)

Source_DATA_Extended Data Fig. 8

Extended Data Fig. 8F: marker

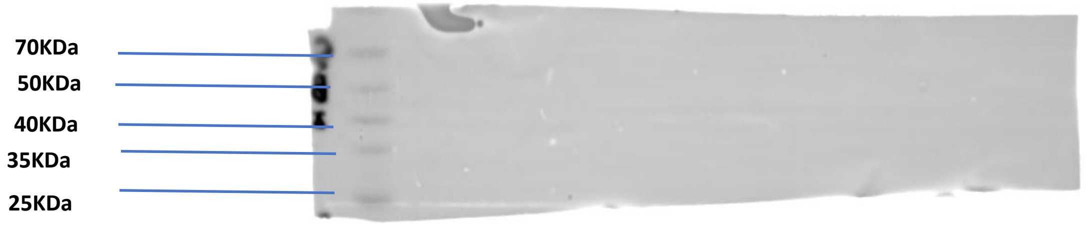

Extended Data Fig. 8F: SERPINB2

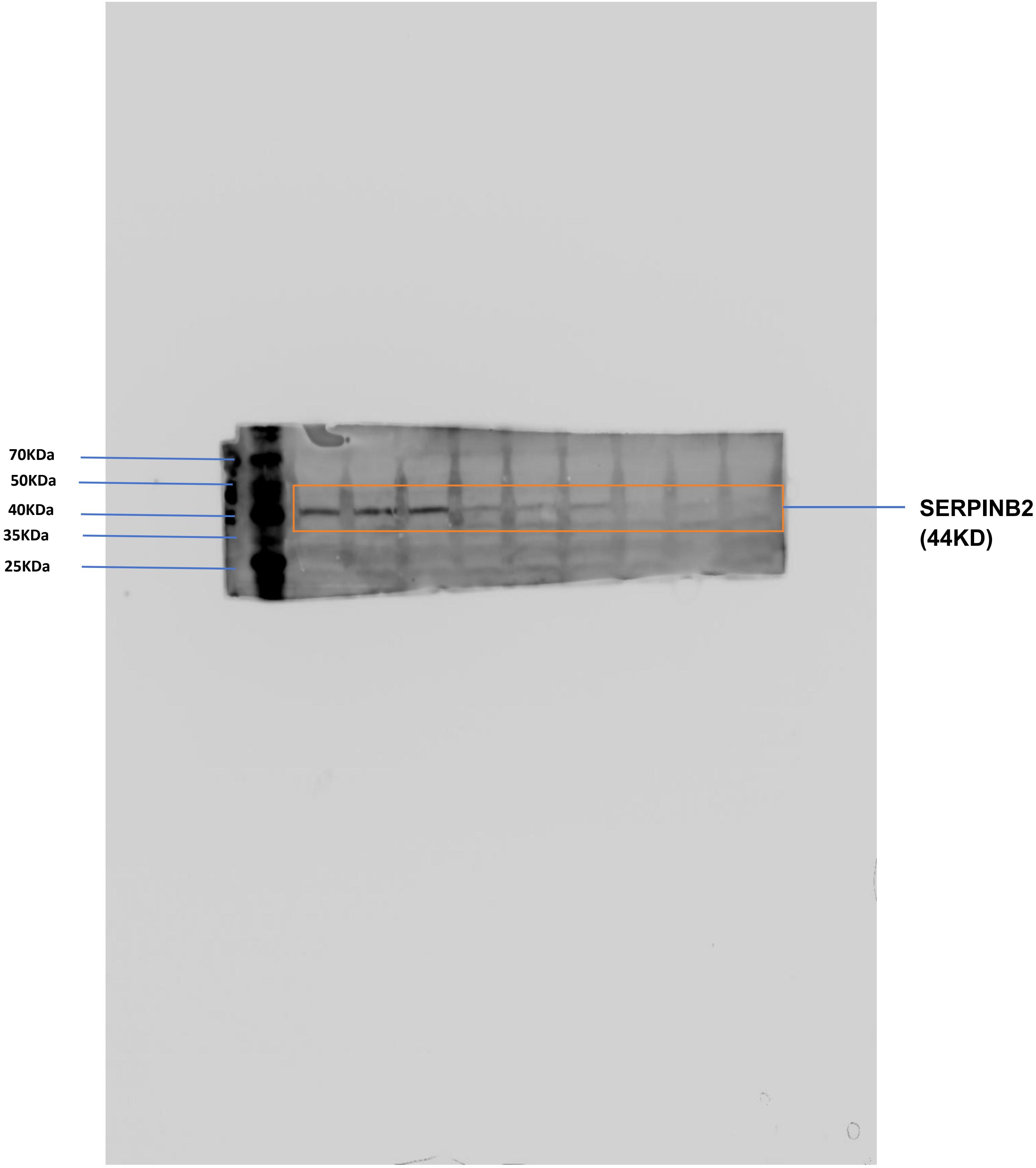

Extended Data Fig. 8F: SERPINB2-sample

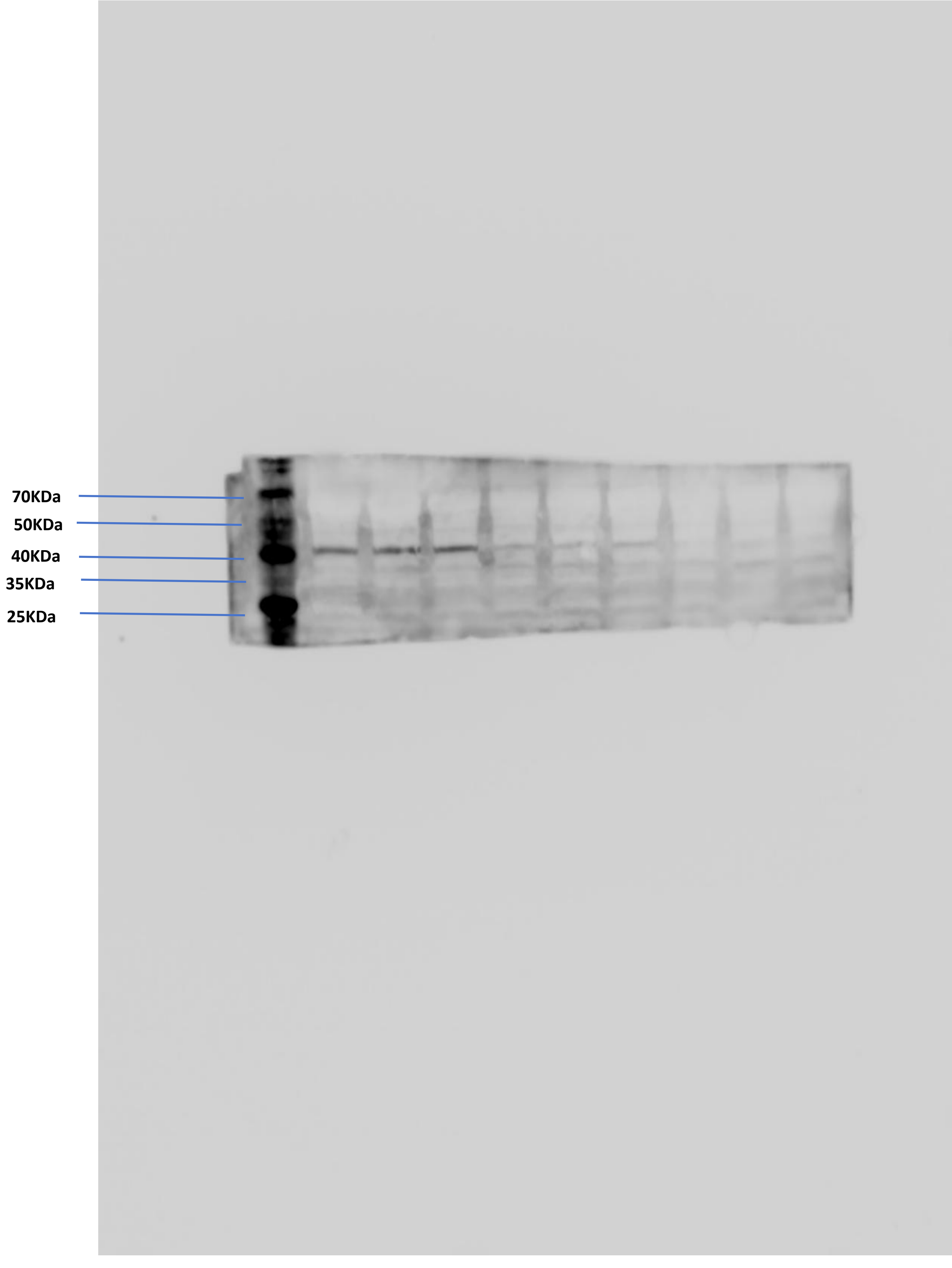

Extended Data Fig. 8F: TNFRSF1A AND TNFSF10

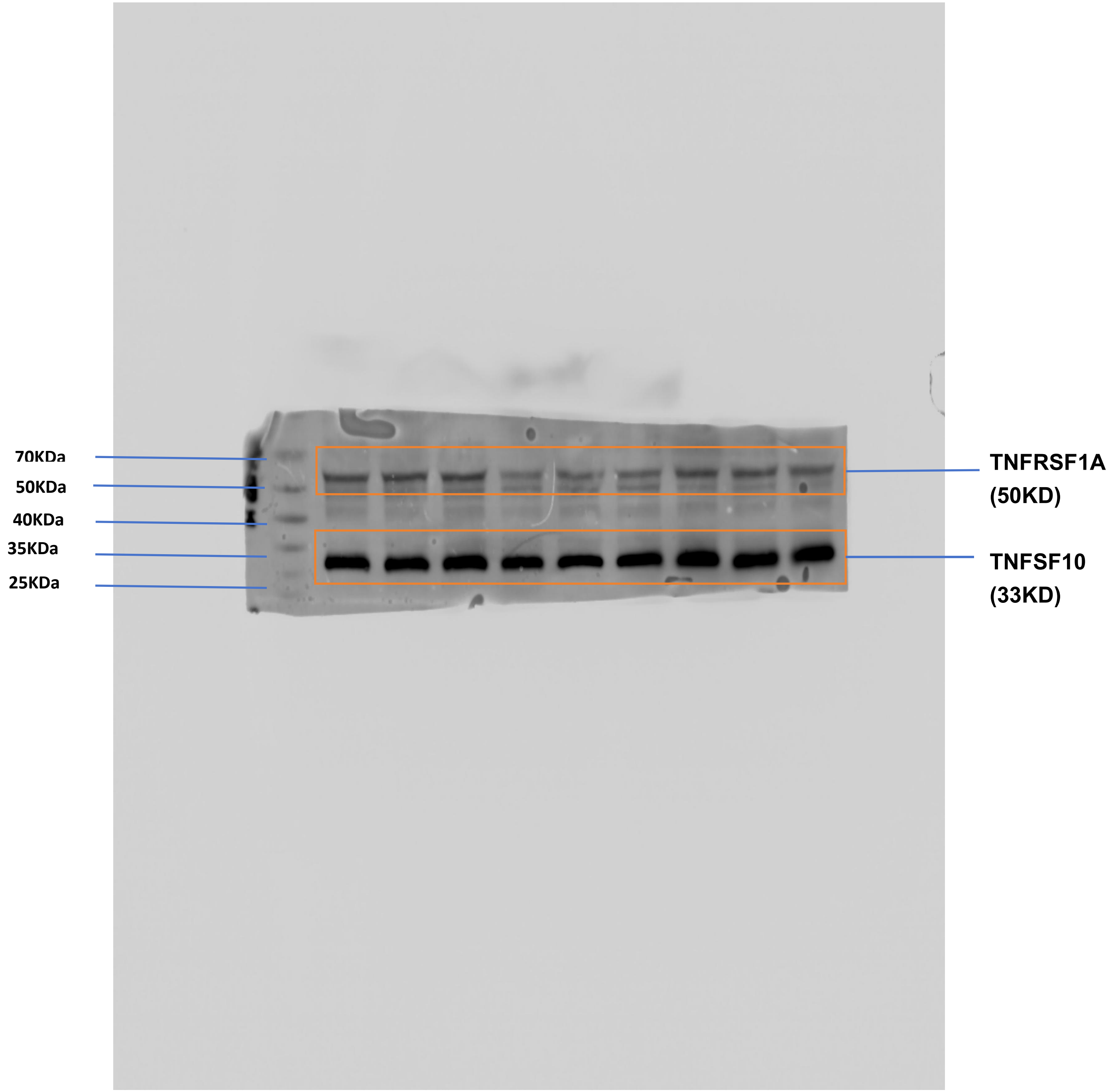

Extended Data Fig. 8F: β -tublin

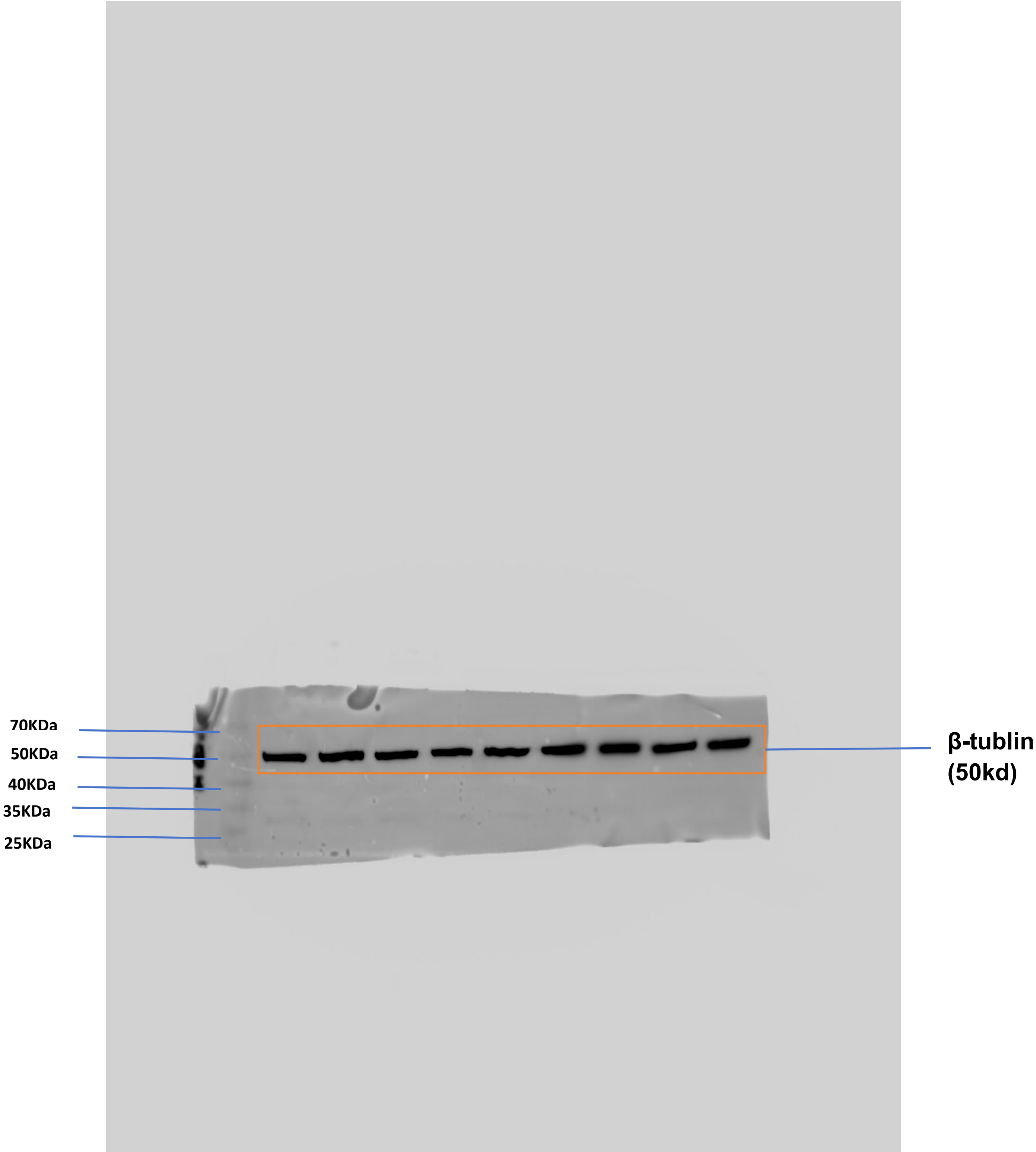

Supplement: Supplementary file 2 [file DataSheet2.pdf]
