# Supplementary material for: Identification of biomarkers for the diagnosis of type 2 diabetes mellitus with metabolic associated fatty liver disease by bioinformatics analysis and experimental validation
Source: Front Endocrinol (Lausanne). 2025 Jan 28;16:1512503. doi: 10.3389/fendo.2025.1512503 (PMC11810736; doi:10.3389/fendo.2025.1512503)
Supplement: Supplementary file 3 [file Table1.docx]

# R related code

rm(list=ls())

Sys.setenv(LANGUAGE = "en")

options(stringsAsFactors = FALSE)

options(BioC_mirror="https://mirrors.ustc.edu.cn/bioc/")

options("repos" = c(CRAN="https://mirrors.tuna.tsinghua.edu.cn/CRAN/"))

library(GEOmirror)

library(AnnoProbe)

library(idmap1)

library(idmap2)

library(idmap3)

getwd()

GSE_ID <- c('GSE89632'，'GSE66676'，'GSE95849'，'GSE22243')

gset<-lapply(GSE_ID,function(GSE_ID){

geoChina(gse=GSE_ID)

})

library(GEOquery)

library(GEOmirror)

library(AnnoProbe)

library(idmap1)

library(idmap2)

library(idmap3)

library(Biobase)

library(utils)

library(plyr)

library(tidyr)

class( gset )

length( gset )

exprSet <- gset[[1]]

str( exprSet, max.level = 2 )

assayData <- exprs(exprSet)

dim(assayData)

class(assayData)

assayData[1:5, 1:6]

phenoData<-pData(exprSet)

dim(phenoData)

dim(phenoData)

head(df)[,1:10]

head(phenoData[,1:5])

table(phenoData$characteristics_ch1.1)

col<-c("title","characteristics_ch1.1")

meta<-phenoData[, col]

table(meta[,2])

gpl <- exprSet@annotation

featureData =get_soft_IDs(gpl)

head(featureData)[,1:5]

head(assayData)[,1:5]

colnames(featureData)

featureData<-featureData[,c("ID","Symbol")]

colnames(featureData)<-c("ID","symbol")

dim(featureData)

featureData <- featureData[featureData$symbol != '', ]##gene

grep("///",featureData$symbol)

index<-intersect(rownames(assayData),featureData$ID)

assayData<-assayData[index,]

rownames(featureData)<-featureData$ID

featureData<-featureData[index,]

identical(rownames(assayData),featureData$ID)

newAssayDate<-assayData

featureData$max <- apply(newAssayDate, 1, max)

featureData[1:15,1:3]

featureData <- featureData[order(featureData$symbol, ##gene

featureData$max,

decreasing = T), ]

featureData <- featureData[featureData $symbol!='',]

dim( featureData )

featureData <- featureData[!duplicated(featureData$symbol), ]

dim( featureData )

colnames(featureData)[2]

ID2gene <- featureData[,1:2]

dim(ID2gene)

AssayData<- newAssayDate[ID2gene$ID,]

dim(AssayData)

AssayData[1:5,1:6]

length(rownames(AssayData))

ID2gene$max<-apply(AssayData,1,max)

ID2gene<-ID2gene[order(ID2gene$symbol,

ID2gene$max,

decreasing=T),]

ID2gene[1:30,1:2]

ID2gene<-ID2gene[!duplicated(ID2gene$symbol),]

dim(ID2gene)

AssayData<-AssayData[ID2gene$ID,]

dim(AssayData)

AssayData[1:5,1:6]

rownames(AssayData) <- ID2gene$symbol

AssayData[1:5, 1:6]

getwd()

library(limma)

library(sva)

rt<-read.csv(file = GSE_file,row.names = 1,check.names = F)

expset<-rt

qx<-as.numeric(quantile(expset,c(0.,0.25,0.5,0.75,0.99,1.0),na.rm=T))

logC<-(qx[5]>100)||

(qx[6]-qx[1]>50&&qx[2]>0)||

(qx[2]>0&&qx[2]<1&&qx[4]>1&&qx[4]<2)

if(logC){expset[which(expset<=0)]<-NaN

expset<-log2(expset)

print("log2 transform finished")}else{print("log2 transform not needed")}

par(cex = 0.7)

n.sample=ncol(expset)

if(n.sample>40) par(cex = 0.5)

cols <- rainbow(n.sample*1.2)

boxplot(expset, col = cols,main="expression value",las=2)

getwd()

expset[1:5,1:5]

expset<-normalizeBetweenArrays(as.matrix(expset,method="scale"))

boxplot(expset, col = cols,main="expression value",las=2)

library(Biobase)

load(GSE_file)

class( gset )

length( gset )

exprSet <- gset[[1]]

phenoData<-pData(exprSet)

dim(phenoData)

library(stringr)

phenoData$characteristics_ch1.1 <- str_replace(phenoData$characteristics_ch1.1, "diagnosis: ","")

phenoData$characteristics_ch1.1[phenoData$characteristics_ch1.1 == "NASH" ] <- "NAFLD"

phenoData$characteristics_ch1.1[phenoData$characteristics_ch1.1 == "SS" ] <- "NAFLD"

phenoData$characteristics_ch1.1[phenoData$characteristics_ch1.1 == "HC" ] <- "Control"

table(phenoData$characteristics_ch1.1)

rownames(phenoData) <- phenoData$X

library(dplyr)

phenoData<- select(phenoData,-X)

cli<-phenoData[,c(11,17)]

colnames(cli)<-c("group","age")

head(cli$age)

table(cli$group)

cli_back<-cli

cli<-cli_back

cli$group<-ifelse(cli$group=="NAFLD"," NAFLD","control")

table(cli$group)

cli<-cli[order(cli$group),]

head(cli)[,1:2]

exp<-exp[,rownames(cli)]

exp2<-t(exp)

do_limma_array <- function(exprSet,group_list){

suppressMessages(library(limma))

design <- model.matrix(~0+factor(group_list))

colnames(design)=levels(factor(group_list))

rownames(design)=colnames(exprSet)

design

#

# dge <- DGEList(counts=exprSet)

# dge <- calcNormFactors(dge)

# logCPM <- cpm(dge, log=TRUE, prior.count=3)

#

# v <- voom(dge,design,plot=TRUE, normalize="quantile")

fit <- lmFit(exprSet, design)

group_list

cont.matrix=makeContrasts(contrasts=c('me-other'),levels = design)

fit2=contrasts.fit(fit,cont.matrix)

fit2=eBayes(fit2)

tempOutput = topTable(fit2, coef='me-other', n=Inf)

DEG_limma = na.omit(tempOutput)

head(DEG_limma)

return(DEG_limma)

}

group_list=ifelse(cli$group=="control",'other','me')

deg1=do_limma_array(exp,group_list)

head(deg1)[,1:5]

library(dplyr)

test<-deg1

test$gene<-rownames(deg1)

test<-arrange(test,test$logFC,test$adj.P.Val)

down25<-test$gene[1:25]

test<-deg1

test$gene<-rownames(deg1)

test<-arrange(test,desc(test$logFC),test$P.Val)

top25<-test$gene[1:25]

head(exp)[,1:5]

heat_exp<-exp[c(down25,top25),]

data<-deg1

data$gene <- rownames(data)

colnames(data)

ml<-"adj.P.Val"

#ml<-"P.Value"

up<-length(data$gene[data[,ml]<0.05&data$logFC >1])

down<-length(data$gene[data[,ml]<0.05&data$logFC< c(-1) ])

deg1$Label = ""

library(tibble)

deg1<-rownames_to_column(deg1,var = "rowname")

deg1<-rownames_to_column(deg1,var = "ID")

deg1$ID<-deg1$rowname

row.names(deg1)<-deg1[,1]

deg1 <- deg1[order(abs(deg1$logFC),decreasing = T), ]

logFC.genes <- head(deg1$ID, 20)

deg1 <- deg1[order(abs(deg1$logFC),decreasing = T), ]

fdr.genes <- head(deg1$ID, 20)

deg.top20.genes <- c(as.character(logFC.genes), as.character(fdr.genes))

deg1$Label[match(deg.top20.genes, deg1$ID)] <- deg.top20.genes

ggplot(data=deg1, aes(x=logFC, y =-log10(adj.P.Val))) +

geom_point(data=subset(data,abs(data$logFC) <= 1),color="#8B8B83",alpha=0.3) +

geom_point(data=subset(data,data[,ml]<0.05 & data$logFC > 1),color="#CD5555",alpha=0.4) +

# geom_point(data=subset(data,data[,ml]<0.05 & data$logFC > 1&data$logFC < 2),aes(size=abs(logFC)),color="#FA8072",alpha=0.4) +

geom_point(data=subset(data,data[,ml]<0.05 & data$logFC < c(-1)),color="darkgreen",alpha=0.4) +

geom_text_repel(

aes(label = Label),

size = 3.2,

color = "black",

segment.color = "black", show.legend = FALSE )+

geom_vline(xintercept = c(1,-1),lty=2,lwd=0.6,alpha=0.8)+

geom_hline(yintercept = c(-log10(0.05)),lty=2,lwd=0.6,alpha=0.8)+

theme_bw()+

ylim(-1,25)+

scale_x_continuous(breaks=seq(-5, 5, 0.5)) +

theme(panel.grid.major = element_blank(),

panel.grid.minor = element_blank(),

axis.text.x = element_text(size = 10,colour = "black"),

axis.text.y = element_text(size = 10,colour = "black"),

axis.title.x = element_text(size = 10,colour = "black"),

axis.title.y = element_text(size = 10,colour = "black"),

axis.line = element_line(colour = "black"))+

labs(x="Log2 (fold change)",y="-log10 (adj.P.Val)")+

theme(legend.position='none')

c2=brewer.pal(9, "Spectral")[c(9,8)]

names(c2)<-na.omit(unique(g$group))

ha= HeatmapAnnotation("Group"=g$group,

annotation_height=unit.c(rep(unit(0.9, "mm"), 2)),

annotation_legend_param=list(labels_gp = gpar(fontsize = 7, fontface = "bold"),

title_gp = gpar(fontsize = 7, fontface = "bold"),

ncol=1),

gap=unit(c(1.1,1.1), "mm"),

col=list("Group"=c2),

na_col = "black",

show_annotation_name = TRUE,

annotation_name_gp = gpar(fontsize = 9))

head(heat_exp)[,1:5]

heat_exp_scale<-as.matrix(t(scale(t(heat_exp))))

heat_exp_scale[heat_exp_scale>c(0.5)]=0.5

heat_exp_scale[heat_exp_scale<c(-0.5)]=c(-0.5)

mypalette <-colorRampPalette(c("lightblue","#ffffff","#FF6A6A"))(300)

ht<- Heatmap(as.matrix(heat_exp_scale),

name="Z-score",

top_annotation = ha,

# right_annotation = ha2,

cluster_rows = T,

clustering_method_rows= "ward.D",

col=mypalette,

color_space = "RGB",

cluster_columns = FALSE,

row_order=NULL,

column_order=NULL,

show_column_names = FALSE,

show_row_names = T,

row_names_gp = gpar(fontsize = 7, fontface = "bold"),

# split=matSplit,

gap = unit(1, "mm"),

# column_title = "TCGA",

column_title_gp = gpar(fontsize = 7, fontface = "bold"),

width=unit(7, "cm"),

show_heatmap_legend = T,

heatmap_legend_param=list(labels_gp = gpar(fontsize = 7),

title_gp = gpar(fontsize = 7)))

draw(ht)

library("WGCNA")

options(stringsAsFactors = FALSE)

femData = read.csv("E:/GSE89632/02_训练集/02_normalized/GSE89632_normalized.csv")

dim(femData)

names(femData)

datExpr0 = as.data.frame(t(femData[,-1]))

names(datExpr0) = femData$X

rownames(datExpr0) = names(femData)[-1]

datExpr0[1:6,1:6]

gsg = goodSamplesGenes(datExpr0, verbose = 3);

gsg$allOK

if(!gsg$allOK)

{

if(sum(!gsg$goodGenes)>0)

printFlush(paste("Removinggenes:",paste(names(datExpr0)[!gsg$goodGenes], collapse =",")));

if(sum(!gsg$goodSamples)>0)

printFlush(paste("Removingsamples:",paste(rownames(datExpr0)[!gsg$goodSamples], collapse =",")));

datExpr0 = datExpr0[gsg$goodSamples, gsg$goodGenes]

}

sampleTree = hclust(dist(datExpr0), method ="average");

sizeGrWindow(18,10)

#pdf(file="Plots/sampleClustering.pdf",width=12,height=9);

par(cex = 0.6)

par(mar =c(4,4,4,4))

plot(sampleTree, main ="Sampleclusteringtodetectoutliers",sub="", xlab="", cex.lab = 1.5,

cex.axis= 1.5, cex.main = 2)

abline(h =140,col="red");

clust = cutreeStatic(sampleTree, cutHeight = 180, minSize = 10)

table(clust)

keepSamples = (clust==1)

datExpr = datExpr0[keepSamples, ]

nGenes =ncol(datExpr)

nSamples =nrow(datExpr)

dim(traitData)

names(traitData)

allTraits = traitData[, -c(24: 26)]

dim(allTraits)

names(allTraits)

femaleSamples =rownames(datExpr)

traitRows =match(femaleSamples, allTraits$X)

datTraits = allTraits[traitRows, -1]

rownames(datTraits) = allTraits[traitRows, 1]

collectGarbage()

sampleTree2 = hclust(dist(datExpr), method ="average")

traitColors = numbers2colors(datTraits, signed = FALSE);

plotDendroAndColors(sampleTree2, traitColors,

groupLabels =names(datTraits),

cex.colorLabels = 0.4, cex.dendroLabels = 0.5,

cex.rowText = 0.6,

marAll = c(1, 5, 3, 1),

main ="Sample dendrogramand trait heatmap")

save(datExpr, datTraits, file = "WGCNA0.3-dataInput.RData")

powers =c(c(1:10),seq(from = 12, to=20,by=2))

powers

sft = pickSoftThreshold(datExpr, powerVector = powers, verbose = 5)

sizeGrWindow(9, 5)

par(mfrow =c(1,2));

cex1 = 0.9;

plot(sft$fitIndices[,1], -sign(sft$fitIndices[,3])*sft$fitIndices[,2],

xlab="SoftThreshold(power)",ylab="ScaleFreeTopologyModelFit,signedR^2",type="n",

main =paste("Scaleindependence"));

text(sft$fitIndices[,1], -sign(sft$fitIndices[,3])*sft$fitIndices[,2],

labels=powers,cex=cex1,col="red");

sft$powerEstimate

abline(h=0.89,col="red")

# Mean Connectivity

plot(sft$fitIndices[,1], sft$fitIndices[,5],

xlab="SoftThreshold(power)",ylab="MeanConnectivity", type="n",

main =paste("Meanconnectivity"))

text(sft$fitIndices[,1], sft$fitIndices[,5],labels=powers, cex=cex1,col="red")

net = blockwiseModules(datExpr,power= 4,

TOMType ="unsigned", minModuleSize = 50,

reassignThreshold = 0, mergeCutHeight = 0.3,

numericLabels = TRUE, pamRespectsDendro = FALSE,

saveTOMs = TRUE,

saveTOMFileBase ="femaleMouseTOM",

verbose = 3)

table(net$colors)

sizeGrWindow(12, 9)

mergedColors = labels2colors(net$colors)

plotDendroAndColors(net$dendrograms[[1]], mergedColors[net$blockGenes[[1]]],

"Modulecolors",

dendroLabels = FALSE, hang = 0.03,

addGuide = TRUE, guideHang = 0.05)

moduleLabels = net$colors

moduleColors = labels2colors(net$colors)

MEs = net$MEs;

geneTree = net$dendrograms[[1]];

save(MEs, moduleLabels, moduleColors, geneTree,

file="FemaleLiver-02-networkConstruction-auto.RData")

nGenes =ncol(datExpr);

nSamples =nrow(datExpr);

MEs0 = moduleEigengenes(datExpr, moduleColors)$eigengenes

MEs = orderMEs(MEs0)

moduleTraitCor =cor(MEs, datTraits, use ="p");

moduleTraitPvalue = corPvalueStudent(moduleTraitCor, nSamples)

table(moduleColors)

textMatrix = paste(signif(moduleTraitCor, 2), "\n(", signif(moduleTraitPvalue, 1), ")", sep = "")

dim(textMatrix) = dim(moduleTraitCor)

sizeGrWindow(9, 9)

par(mar = c(3, 8, 3, 3))

labeledHeatmap(Matrix = moduleTraitCor,

xLabels =names(datTraits),

yLabels =names(MEs),

ySymbols =names(MEs),

colorLabels = FALSE,

colors= blueWhiteRed(50),

textMatrix = textMatrix,

setStdMargins = FALSE,

cex.text= 0.4,

cex.lab = 0.9,

zlim =c(-1,1),

main =paste("Module-traitrelationships"))

dev.off()

allLLIDs = annot$X;

intModules = c('brown','blue')

for (module in intModules)

{

modGenes = (moduleColors==module)

modLLIDs = allLLIDs[modGenes];

fileName = paste("LocusLinkIDs-", module, ".txt", sep="");

write.table(as.data.frame(modLLIDs), file = fileName,

row.names = FALSE, col.names = FALSE)

}

library (VennDiagram)

library(openxlsx)

T2DM<-read.xlsx('T2DM-nafld.xlsx',sheet= 'T2DM',sep=',')

NAFLD<-read.xlsx('T2DM-nafld.xlsx',sheet= "NAFLD",sep=',')

T2DM=t(T2DM)

NAFLD=t(NAFLD)

head(T2DM)

venn.diagram(x=list(NAFLD,T2DM),

scaled = F,

alpha= 0.5,

lwd=1,lty=1,col=c('cadetblue1',"lightcoral"),

label.col ='black' ,

cex = 2,

fontface = "bold",

fill=c('green',"blue"),

category.names = c("NAFLD", "T2DM relative secretory protein") ,

cat.dist = 0.02,

cat.pos = -180,

cat.cex = 1.5,

cat.fontface = "bold",

cat.col='black' , #cat.col=c('#FFFFCC','#CCFFFF',.....)

cat.default.pos = "outer",

output=TRUE,

imagetype="tiff",

resolution = 400,

compression = "lzw"

)

grid.draw(data)

data.list<-list(T2DM=na.omit(T2DM),NAFLD=na.omit(NAFLD))

inter <- get.venn.partitions(data.list)

write.xlsx(inter,"inter_result.xlsx")

library(openxlsx)

library(ggplot2)

library(enrichplot)

library(clusterProfiler)

library(GOplot)

library(DOSE)

library(ggnewscale)

library(topGO)

library(circlize)

library(ComplexHeatmap)

info <- read.table("HUB.txt", quote="\"", comment.char="")

GO_database <- 'org.Hs.eg.db'

KEGG_database <- 'hsa'

gene <- bitr(info$V1,fromType = 'SYMBOL',toType = 'ENTREZID',OrgDb = GO_database)

GO<-enrichGO( gene$ENTREZID,

OrgDb = GO_database,

keyType = "ENTREZID",

ont = "ALL",

pvalueCutoff = 0.05,

qvalueCutoff = 0.05,

readable = T)

options(clusterProfiler.download.method = "wininet")

KEGG<-enrichKEGG(gene$ENTREZID,

organism = KEGG_database,

pvalueCutoff = 0.05,

qvalueCutoff = 0.05

)

KEGG <- setReadable(KEGG, OrgDb = org.Hs.eg.db, keyType="ENTREZID")

library(org.Hs.eg.db)

barplot(GO, split="ONTOLOGY",showCategory = 10,label_format=50)+facet_grid(ONTOLOGY~., scale="free")

barplot(KEGG,showCategory = 40,label_format=80,title = 'KEGG Pathway')

dotplot(GO, split="ONTOLOGY",label_format=50)+facet_grid(ONTOLOGY~., scale="free")

dotplot(KEGG,label_format=80)

options(ggrepel.max.overlaps=Inf)

enrichplot::cnetplot(GO,circular=TRUE,color.params = list(edge = 60,category_node =0.2,gene_label = 0.2), force = 1,max.overlaps =300

enrichplot::cnetplot(KEGG,circular=TRUE,color.params = list(edge = 50),force = 1,node_label = "all",category_node =0.2,gene_label = 0.2)

enrichplot::heatplot(GO,showCategory = 30)

enrichplot::heatplot(KEGG,showCategory = 50)

library(glmnet)

library(marray)

library(stringr)

library(caret)

library(survminer)

library(survival)

ind<-read.table("input.txt", quote="\"", comment.char="")

table(cli$group)

rt<-exp[ind$V1,]

rt=t(rt)

rt<-as.data.frame(rt)

x=as.matrix(rt)

y = cli$group

fit=glmnet(x, y, family = "binomial", alpha=1)

pdf("lambda.pdf")

plot(fit, xvar = "lambda", label = TRUE)

dev.off()

cvfit=cv.glmnet(x, y, family="binomial", alpha=1,type.measure='deviance',nfolds = 10)

pdf(file="cvfit.pdf",width=6,height=5.5)

plot(cvfit)

dev.off()

cvfit$lambda.min

coef=coef(fit, s = cvfit$lambda.min)

index=which(coef != 0)

lassoGene=row.names(coef)[index]

lassoGene=lassoGene[-1]

write.table(lassoGene, file="LASSO.gene.txt", sep="\t", quote=F, row.names=F, col.names=F)

rt1=t(rt)

lassoexp=rt1[lassoGene,,drop=F]

lassoexp=as.data.frame(lassoexp)

write.table(lassoexp, file="LASSO.geneExp.txt", sep="\t", quote=F, row.names=T, col.names=T)

library(tidyverse)

library(glmnet)

library(VennDiagram)

library(sigFeature)

library(e1071)

library(caret)

library(randomForest)

library(bRacatus)

source("E:/GSE15653/svf/msvmRFE.R")

quote="\"", comment.char="")

input <- read.delim("svf/input2.txt")

row.names(input)<- input$X

library(dplyr)

input <- dplyr::select(input,-X)

input$group

svmRFE(input,k=10,halve.above=10)

nfold = 10

nrows = nrow(input)

folds = rep(1:nfold, len=nrows)[sample(nrows)]

folds = lapply(1:nfold, function(x) which(folds == x))

results = lapply(folds, svmRFE.wrap, input, k=10, halve.above=100)

top.features = WriteFeatures(results, input, save=F)

write.table(top.features, file="top.features.txt", sep="\t", quote=F, row.names=T, col.names=T)

featsweep = lapply(1:5, FeatSweep.wrap, results, input)

save(featsweep,file = "featsweep.RData")

no.info = min(prop.table(table(input[,1])))

errors = sapply(featsweep, function(x) ifelse(is.null(x), NA, x$error))

dev.new(width=6,height=4,bg='white')

pdf("svm_error.pdf", height = 5, width = 10)

PlotErrors(errors, no.info=no.info)

dev.off()

plot(top.features)

pdf("6B_svm-accuracy.pdf",width = 5,height = 5)

Plotaccuracy(1-errors,no.info=no.info,)

dev.off()

which.min(errors)

top<-top.features[1:which.min(errors), "FeatureName"]

write.csv(top,"top.csv")

library(randomForest)

set.seed(123456)

ind<-read.table("input.txt", quote="\"", comment.char="")

table(cli$group)

data<-exp[ind$V1,]

data=t(data)

data=as.data.frame(data)

group<-cli$group

rf=randomForest(as.factor(group)~., data=data, ntree=1000)

pdf(file="森林.pdf", width=6, height=6)

plot(rf, main="Random forest", lwd=2)

dev.off()

optionTrees=which.min(rf$err.rate[,1])

optionTrees

rf2=randomForest(as.factor(group)~., data=data, ntree=optionTrees)

importance=importance(x=rf2)

pdf(file="GeneIm.pdf", width=6.2, height=9)

varImpPlot(rf2, main="")

dev.off()

rfGenes=importance[order(importance[,"MeanDecreaseGini"], decreasing = TRUE),]

#rfGenes=names(rfGenes[rfGenes>2])

rfGenes=names(rfGenes[rfGenes>1])

#rfGenes=names(rfGenes[1:30])

write.table(rfGenes, file="随机森林Genes1.txt", sep="\t", quote=F, col.names=F, row.names=F)

sigExp=t(data[,rfGenes])

sigExpOut=rbind(ID=colnames(sigExp),sigExp)

write.table(sigExpOut, file="imGeneExp2.txt", sep="\t", quote=F, col.names=F)

write.table(cli, file="cli.txt", sep="\t", quote=F, row.names=T, col.names=T)

write.table(exp, file="exp.txt", sep="\t", quote=F, row.names=T, col.names=T)

library (VennDiagram)

library(openxlsx)

LASSO<-read.xlsx('3.xlsx',sheet= "LASSO",sep=',')

RF<-read.xlsx('3.xlsx',sheet= "RF",sep=',')

SVF<-read.xlsx('3.xlsx',sheet= "SVF",sep=',')

LASSO=t(LASSO)

RF=t(RF)

SVF=t(SVF)

head(SVF)

venn.diagram(x=list(LASSO,RF,SVF),

scaled = F,

alpha= 0.5,

lwd=1,lty=1,col=c('#FFFFCC','#CCFFFF',"#FFCCCC"),

label.col ='black' , # 数字颜色abel.col=c('#FFFFCC','#CCFFFF',......)

cex = 2,

fontface = "bold",

fill=c('#FFFFCC','#CCFFFF',"#FFCCCC"),

category.names = c("LASSO", "RF","SVF-RM") ,

cat.dist = 0.02,

cat.pos = c(-10, 10, 135), -240, -180

cat.cex = 1,

cat.fontface = "bold",

cat.col='black' , #cat.col=c('#FFFFCC','#CCFFFF',.....)

cat.default.pos = "outer",

output=TRUE,

filename='三组.tiff',

imagetype="tiff",

resolution = 600,

compression = "lzw"

)

grid.draw(data)

data.list<-list(LASSO,RF,SVF)

inter <- get.venn.partitions(data.list)

write.xlsx(inter,"inter_result.xlsx")

rm(list=ls())

Sys.setenv(LANGUAGE = "en") #显示英文报错信息

options(stringsAsFactors = FALSE) #禁止chr转成factor

options("repos" = c(CRAN="https://mirrors.tuna.tsinghua.edu.cn/CRAN/"))

library(rms)

library(survival)

library(tidyverse)

data<- read.table("inputFile.txt",sep= "\t" ,header = T)

gbsg<-data

head(gbsg)

str(gbsg)

##gbsg=as.data.frame(lapply(gbsg,as.integer))

ddist <- datadist(gbsg)

options(datadist='ddist')

# 拟合逻辑回归模型

model <- lrm(Type ~ ., data = gbsg,x = T, y = T)

fit<-model

cbind(coef=coef(fit),OR=exp(coef(fit)))

nomogram <- nomogram(model, fun = function(x)1/(1+exp(-x)),funlabel="Risk of NAFLD",conf.int=F,lp=F,fun.at=c(0.01,0.15,0.5,0.85,0.99))

plot(nomogram)

library(PredictABEL)

cal1 <- calibrate(fit, method = 'boot', B = 100)

plot(cal1,xlim = c(0,1.0),ylim = c(0,1.0)) #method = 'boot', B = 100

library(rmda)

gbsg<-as.data.frame(gbsg)

simple_TNFSF10<-decision_curve(Type ~ TNFSF10,

data = gbsg,family = binomial(link ='logit'),

thresholds = seq(0,1, by = 0.01),

confidence.intervals= 0.95,

study.design = 'case-control',

population.prevalence = 0.3)

simple_SERPINB2<-decision_curve(Type~ SERPINB2,

data = gbsg,family = binomial(link ='logit'),

thresholds = seq(0,1, by = 0.01),

confidence.intervals= 0.95,

study.design = 'case-control',

population.prevalence = 0.3)

simple_TNFRSF1A<-decision_curve(Type ~ TNFRSF1A,

data = gbsg,family = binomial(link ='logit'),

thresholds = seq(0,1, by = 0.01),

confidence.intervals= 0.95,

study.design = 'case-control',

population.prevalence = 0.3)

complex<-decision_curve(Type ~ TNFSF10 + SERPINB2 + TNFRSF1A,

data = gbsg,family = binomial(link ='logit'),

thresholds = seq(0,1, by = 0.01),

confidence.intervals= 0.95,

study.design = 'case-control',

population.prevalence = 0.3)

List<- list(simple_TNFSF10,simple_SERPINB2,simple_TNFRSF1A,complex)

plot_decision_curve(List,

curve.names=c('TNFSF10', 'SERPINB2','TNFRSF1A','complex'),

cost.benefit.axis =FALSE,col= c("#0072B2", "#E69F00", "#009E73", "#D55E33", "#CC79A7","blue"),

confidence.intervals=FALSE,

standardize = FALSE)

library(plyr)

library(rms)

library(epiDisplay)

library(gtsummary)

aa<- read.table("inputFile.txt",sep= "\t" ,header = T)

names(aa)

str(aa)

#for (i in names(aa)[c(1,4:12)]){aa[,i] <- as.factor(aa[,i])}

Uni_glm_model<-

function(x){

FML<-as.formula(paste0("Type==0~",x))

glm1<-glm(FML,data=aa,family = binomial,control = list(maxit = 100))

glm2<-summary(glm1)

OR<-round(exp(coef(glm1)),2)

SE<-glm2$coefficients[,2]

CI5<-round(exp(coef(glm1)-1.96*SE),2)

CI95<-round(exp(coef(glm1)+1.96*SE),2)

CI<-paste0(CI5,'-',CI95)

P<-round(glm2$coefficients[,4],2)

Uni_glm_model <- data.frame('Characteristics'=x,

'OR' = OR,

'CI' = CI,

'P' = P)[-1,]

return(Uni_glm_model)

}

variable.names<- colnames(aa)[c(2:6)];variable.names

Uni_glm <- lapply(variable.names, Uni_glm_model)

library(plyr)

Uni_glm<- ldply(Uni_glm,data.frame);Uni_glm

variable.names

names<- glm(status==0~age+race+marry+t+n+tnm+er+pr+her2+g+sur+rt+che,

data=aa,control = list(maxit = 100),

family = binomial)

name<-data.frame(summary(names)$aliased)

rownames(Uni_glm)<-rownames(name)[-1]

Uni_glm <- Uni_glm[,-1]

library(RColorBrewer)

library(ggpubr)

library(ggplot2)

library(cowplot)

rt=read.delim("input.txt")

ind<-read.delim("info.txt", header=FALSE)

#cli$group<-ifelse(cli$group==" NAFLD",1,0)

ind<-as.character(ind)

data<-rt[,ind]

write.table(data, file="data.txt", sep="\t", quote=F, row.names=T, col.names=T)

rt=read.delim("inputFile.txt")

HUB<-read.delim("HUB.txt", header=FALSE)

gene<- as.vector(HUB$V1)

rt$Type<- as.factor(rt$Type)

Exp_plot <- rt

col <-c("#5CB85C","#337AB7")

plist2<-list()

for (i in 1:length(gene)){

bar_tmp<-Exp_plot[,c(gene[i],"Type")]

colnames(bar_tmp)<-c("Expression","Type")

my_comparisons1 <- list(c("NAFLD", "control"))

pb1<-ggboxplot(bar_tmp,

x="Type",

y="Expression",

color="Type",

fill=NULL,

add = "jitter",

bxp.errorbar.width = 0.6,

width = 0.4,

size=0.01,

font.label = list(size=28),

palette = col)+theme(panel.background =element_blank())

pb1<-pb1+theme(axis.line=element_line(colour="black"))+theme(axis.title.x = element_blank())

pb1<-pb1+theme(axis.title.y = element_blank())+theme(axis.text.x = element_text(size = 15,angle = 45,vjust = 1,hjust = 1))

pb1<-pb1+theme(axis.text.y = element_text(size = 15))+ggtitle(gene[i])+theme(plot.title = element_text(hjust = 0.5,size=10,face="bold"))

pb1<-pb1+theme(legend.position = "NA")#

pb1<-pb1+stat_compare_means(method="wilcox.test",hide.ns = F,

comparisons =c(my_comparisons1),

label="p.signif")

plist2[[i]]<-pb1

}

plot_grid(plist2[[1]],plist2[[2]],plist2[[3]],

ncol=3

Sys.setenv(LANGUAGE = "en") source("CIBERSORT.R")

load(file = "Normal.Rdata")

LM22.file <- "LM22.txt"

exp.file <- "datExp.processed.txt"

TME.results = CIBERSORT(LM22.file, exp.file, perm = 1000, QN = TRUE)

write.table(TME.results, "TME.results.output.txt",

sep = "\t", row.names = T, col.names = T, quote = F)

results<-read.table("TME.results.output.txt",header=TRUE,row.names = 1,check.names = FALSE,sep="\t")

results[1:5,]

library(pheatmap)

re <- results[,-(23:25)]

k <- apply(re,2,function(x) {sum(x == 0) < nrow(results)/2})

re2 <- as.data.frame(t(re[,k]))

cli$Sample <- rownames(cli)

an = data.frame(Group = cli$group,

row.names = cli$Sample)

bk <- c(seq(-15,-5,by=1),seq(-4.9,4.9,by=0.2),seq(5,15,by=1))

pheatmap(re2,scale = "row",

show_colnames = F,

cluster_cols = F,

annotation_col = an,

drop_levels = TRUE,

color = c(rep("blue",11),colorRampPalette(colors = c("blue","white","red"))(50),

rep("red",11)),

breaks = bk,

#legend_breaks = c(-5,-2,0,2,5)

)

##########直方图

library(RColorBrewer)

library(tidyr)

library(ggplot2)

library(tibble)

mypalette <- colorRampPalette(brewer.pal(8,"Set1"))

dat <- re %>% as.data.frame() %>%

rownames_to_column("Sample") %>%

gather(key = Cell_type,value = Proportion,-Sample)

dev.new()

ggplot(dat,aes(Sample,Proportion,fill = Cell_type)) +

geom_bar(stat = "identity") +

labs(fill = "Cell Type",x = "",y = "Estiamted Proportion") +

theme_bw() +

theme(axis.text.x = element_blank(),

axis.ticks.x = element_blank(),

legend.position = "bottom") +

scale_y_continuous(expand = c(0.01,0)) +

scale_fill_manual(values = mypalette(22))

ggplot(dat,aes(Cell_type,Proportion,fill = Cell_type)) +

geom_boxplot(outlier.shape = 21,color = "black") +

theme_bw() +

labs(x = "Cell Type", y = "Estimated Proportion") +

theme(axis.text.x = element_blank(),

axis.ticks.x = element_blank(),

legend.position = "bottom") +

scale_fill_manual(values = mypalette(22))

library(dplyr)

a = dat %>%

group_by(Cell_type) %>%

summarise(m = median(Proportion)) %>%

arrange(desc(m)) %>%

pull(Cell_type)

dat$Cell_type = factor(dat$Cell_type,levels = a)

ggplot(dat,aes(Cell_type,Proportion,fill = Cell_type)) +

geom_boxplot(outlier.shape = 21,color = "black") +

theme_bw() +

labs(x = "Cell Type", y = "Estimated Proportion") +

theme(axis.text.x = element_blank(),

axis.ticks.x = element_blank(),

legend.position = "bottom") +

scale_fill_manual(values = mypalette(22))

library(stringr)

library(ggpubr)

dat$Group = ifelse(as.numeric(str_sub(dat$Sample,14,15))<10,"NAFLD","normal")

dat$Group =an$Group

ggplot(dat,aes(Cell_type,Proportion,fill = Group)) +

geom_boxplot(outlier.shape = 21,color = "black") +

theme_bw() +

labs(x = "Cell Type", y = "Estimated Proportion") +

theme(legend.position = "top") +

theme(axis.text.x = element_text(angle=80,vjust = 0.5))+

scale_fill_manual(values = mypalette(22)[c(6,1)])+

stat_compare_means(aes(group = Group,label = ..p.signif..),method = "kruskal.test")

source("CIBERSORT.R")

library(ggplot2)

library(reshape2)

library(ggpubr)

library(tidyverse)

library(RColorBrewer)

library(corrplot)

library(ggsci)

load(file = "Normal.Rdata")

LM22.file <- "LM22.txt"

exp.file <- "datExp.processed.txt"

TME.results = CIBERSORT(LM22.file, exp.file, perm = 1000, QN = TRUE)

write.table(TME.results, "TME.results.output.txt",

sep = "\t", row.names = T, col.names = T, quote = F)

results<-read.table("TME.results.output.txt",header=TRUE,row.names = 1,check.names = FALSE,sep="\t")

results[1:5,]

cibersort_data <- as.data.frame(results[,1:22])

cibersort_data<-rownames_to_column(cibersort_data,var="Sample")

cli$Sample <- rownames(cli)

cli<-as.data.frame(cli[,-2])

Group<-cli

cibersort<-left_join(cibersort_data,Group,by="Sample")

cibersort<- melt(cibersort,id.vars=c("Sample","group"))

colnames(cibersort)<-c("Sample","Group","celltype","composition")

exp<-read.table("exp.txt",header=T,row.names=1,sep="\t")

genelist<-c("TNFSF10","SERPINB2","TNFRSF1A")

goal_exp<-filter(exp,rownames(exp) %in%genelist)

X<-t(cibersort_data)

colnames(X)<-cibersort_data$Sample

X<-as.data.frame(X[-1,])

combine<-rbind(goal_exp,as.data.frame(X))

D<-as.data.frame(t(combine))

write.csv(D, "D.csv", row.names = TRUE, quote = TRUE)

D <- read.csv("D.csv", header = TRUE, row.names=1,stringsAsFactors = F)

comcor<-cor(D)

comp<-cor.mtest(comcor,conf.level=0.95)

pval<-comp$p

goalcor<-select(as.data.frame(comcor),genelist)%>%rownames_to_column(var="celltype")

goalcor<-filter(goalcor,!(celltype %in% genelist))

goalcor<-melt(goalcor,id.vars="celltype")

colnames(goalcor)<-c("celltype","Gene","correlation")

pval<-select(as.data.frame(pval),genelist)%>%rownames_to_column(var="celltype")

pval<-filter(pval,!(celltype %in% genelist))

pval<-melt(pval,id.vars="celltype")

colnames(pval)<-c("celltype","gene","pvalue")

final<-left_join(goalcor,pval,by=c("celltype"="celltype","Gene"="gene"))

final$sign<-case_when(final$pvalue<0.05 &final$pvalue>0.01 ~"*",

final$pvalue<0.01 &final$pvalue>0.001 ~"**",

final$pvalue<0.001 ~"***",

final$pvalue>0.05 ~"")

ggplot(data=final,aes(x=Gene,y=celltype))+

geom_tile(aes(fill=correlation),colour="white",size=1)+

scale_fill_gradient2(low="#2b8cbe",mid="white",high="#e41a1c")+

geom_text(aes(label=sign),colour="black")+

theme_minimal()+

theme(axis.text.x=element_text(angle=45,hjust=1,size=12),

axis.text.y=element_text(size=12),

axis.title.x=element_blank(),

axis.title.y=element_blank(),

axis.ticks.x=element_blank(),

axis.ticks.y=element_blank()) +

guides(fill=guide_legend(title="* p<0.05\n\n** p<0.01\n\n*** p<0.001\n\ncorrelation"))

ggsave("correlation.pdf",width=8,height=8)

write.csv(cibersort_data, "cibersort_data.csv", row.names = TRUE, quote = TRUE)

cibersort_data<- read.csv("cibersort_data.csv", header = TRUE, stringsAsFactors = F)

rownames(cibersort_data)<-cibersort_data$X

cibersort_data<-as.data.frame(results[,1:22])

cor<-cor(cibersort_data)

pdf('corplot.pdf',height=14,width=14)

corrplot(cor,method=c('number'))

dev.off()
